# METTL3/m6A‐Dependent SERPINE1/VEGFA Axis Mediates Sublethal Heat‐Induced Angiogenesis in Hepatocellular Carcinoma

**DOI:** 10.1155/mi/5133850

**Published:** 2026-03-24

**Authors:** Zhuoyang Fan, Yang Gao, Juncheng Wan, Yiwei Zhu, Ming Li, Xiaochen Chen, Guowei Yang

**Affiliations:** ^1^ Department of Interventional Radiology, Zhongshan Hospital, Fudan University, Shanghai, 200032, China, fudan.edu.cn; ^2^ National Clinical Research Center for Interventional Medicine, Zhongshan Hospital, Fudan University, Shanghai, China, fudan.edu.cn; ^3^ Shanghai Institute of Medical Imaging, Shanghai, China; ^4^ Department of Neurosurgery, Zhongshan Hospital, Fudan University, Shanghai, 200032, China, fudan.edu.cn; ^5^ Shanghai World Foreign Language Academy, Shanghai, 200032, China; ^6^ Department of Thoracic Surgery, Zhongshan Hospital, Fudan University, Shanghai, 200032, China, fudan.edu.cn; ^7^ Clinical Research Institute, Shanghai Jiao Tong University School of Medicine, Shanghai, 200025, China, shsmu.edu.cn

**Keywords:** angiogenesis, heat ablation, hepatocellular carcinoma, m6A, SERPINE1

## Abstract

Heat ablation techniques, such as microwave and radiofrequency ablation, are established interventions for hepatocellular carcinoma (HCC). However, incomplete ablation often leads to angiogenesis‐driven metastasis and recurrence, undermining long‐term treatment efficacy. The molecular mechanisms facilitating post‐ablation angiogenesis remain poorly understood. In this study, we identified a marked upregulation of SERPINE1 following sublethal heat treatment, which was corroborated in both rabbit models and human HCC cell lines. Further investigation revealed that SERPINE1 promotes angiogenesis, at least in part, through vascular endothelial growth factor A (VEGFA) activation after sublethal heat exposure. We further delineated that METTL3 and IGF2BP1 regulate SERPINE1 expression via an N6‐methyladenosine (m6A)‐dependent pathway. The proangiogenic role of SERPINE1 was substantiated using patient‐derived HCC organoids and in vivo models, where the small‐molecule inhibitor PAI‐039 significantly attenuated sublethal heat ablation‐induced angiogenesis and tumor proliferation. Our findings illuminate the METTL3/IGF2BP1/SERPINE1/VEGFA axis as a novel therapeutic target for improving HCC heat ablation outcomes. Therapeutically, PAI‐039 emerges as a potent adjunctive agent that could synergistically enhance the efficacy of thermal ablation in HCC management.

## 1. Introduction

Hepatocellular carcinoma (HCC) is a leading cause of cancer‐related death worldwide, with an estimated 1,000,000 new cases projected for 2025 [1, 2]. Conventional treatment strategies include surgical resection, interventional therapy, and thermal ablation (e.g., microwave or radiofrequency ablation). Microwave ablation, endorsed by clinical guidelines, is an indispensable modality for HCC eradication [3]. Nonetheless, metastasis and recurrence of residual tumor cells following incomplete (sublethal) ablation remain major causes of treatment failure.

Plasminogen activator inhibitor type 1 (SERPINE1/PAI‐1), a member of the serine protease inhibitor superfamily, was initially characterized as an inhibitor of matrix metalloproteinase (MMP) activation, thereby disrupting extracellular matrix homeostasis [4]. SERPINE1 is closely implicated in tumor pathogenesis. For instance, McCann et al. [5] reported that miR‐30c inhibits carcinogenesis by blocking SERPINE1 in breast and lung cancers, while Teng et al. [6] demonstrated that the NKX2‐1‐AS1/miR‐145‐5p axis upregulates SERPINE1 translation in gastric cancer. However, the precise role and regulatory mechanism of SERPINE1 in HCC, particularly in the context of thermal ablation, are not fully elucidated.

Thermal ablation induces significant remodeling of the tumor microenvironment (TME) in HCC [7, 8]. SERPINE1 has been reported to contribute to the immune TME [9] and promote colon cancer progression by TME remodeling [10], underscoring its importance in the tumor stroma. Angiogenesis is a critical pathological process driving tumor metastasis and recurrence [11]. HCC is characterized by abundant and aberrant vasculature, which aids in clinical diagnosis [12] but also renders antiangiogenic therapy a viable treatment strategy. Vascular endothelial growth factor A (VEGFA) is a pivotal proangiogenic cytokine [13], and its tumor‐promoting role in HCC is well‐documented [14–16]. Although the multikinase inhibitor sorafenib, which targets VEGFRs, has been a standard treatment for advanced HCC for over a decade [17], it only extends overall survival by several months [18], highlighting the need for novel therapeutic targets.

RNA methylation, a key posttranscriptional modification in eukaryotes, regulates diverse physiological processes [19]. N6‐methyladenosine (m6A) is the most prevalent mRNA modification and is extensively involved in tumor metastasis. For example, METTL3/m6A promotes esophageal squamous cell carcinoma progression via GLS2 [20], YTHDC1/m6A suppresses pancreatic ductal adenocarcinoma by upregulating miR‐30d [21], and m6A modification status influences breast cancer lung metastasis [22]. However, whether and how m6A modification regulates angiogenesis following sublethal heat treatment in HCC remains unknown.

Patient‐derived organoids (PDOs) have emerged as powerful tools in cancer research, more accurately recapitulating tumor histology and genetics than traditional 2D cell lines [23, 24]. As intermediate 3D in vitro models, PDOs self‐renew, self‐organize, and retain key features of the original tumor [25]. In our previous work, we used HCC PDOs to model sublethal heat treatment and identified CD47‐mediated epithelial‐mesenchymal transition (EMT) as a prometastatic mechanism [26]. These findings laid the groundwork for the current study.

In summary, while sublethal heat treatment is known to promote HCC invasion [26], and angiogenesis is tightly linked to metastasis [27, 28], the connecting molecular mechanisms are unclear. Here, we investigated the role of SERPINE1 in sublethal heat‐induced angiogenesis beyond its known functions in the TME, proposing it as a key therapeutic target to inhibit post‐ablation HCC metastasis.

## 2. Methods

### 2.1. Cell Culture and Treatment

In this project, we used two human HCC cell lines, HCCLM3 and Huh7, to perform the in vitro study, and used one rabbit squamous cell line, VX2, to carry out the rabbit in situ transplantation. The three cell lines were obtained from the Liver Cancer Institute, Zhongshan Hospital, Fudan University (Shanghai, China). VX2 cell has been widely used as a classic liver tumor model [29–31]. Cells were cultured in Dulbecco’s modified Eagle’s medium (DMEM, Gibco, Grand Island, NY, USA), which contained 10% fetal bovine serum (FBS) and antibiotics (penicillin [100 U/mL]/streptomycin [0.1 mg/mL]). The cells were incubated at 37°C and 5% CO_2_ in a humidified environment, and the medium was refreshed three times a week.

Information on the cell lines in the following sections:

#### 2.1.1. HCCLM3

• Species: *Homo sapiens* (human).
• Sex: Male (derived from male patient).
• Tissue origin: Primary HCC, metastatic lesion in lung (established from LCI‐D20 tumor model).
• Official cell line name: HCCLM3 (high metastatic potential subclone of MHCC97‐H).
• RRID: CVCL_6830 (Cellosaurus database).

• Key characteristics are as follows:

    High metastatic potential (established via in vivo selection).

    Expresses HCC markers (AFP and GPC3) and EMT markers.

    Source: the Liver Cancer Institute, Zhongshan Hospital, Fudan University (Shanghai, China).

#### 2.1.2. Huh7

• Species: *Homo sapiens* (human).
• Sex: Male (derived from male patient).
• Tissue origin: Primary HCC.
• Official cell line name: Huh7.
• RRID: CVCL_0336 (Cellosaurus database).
• Key characteristics are as follows:

    Well‐differentiated HCC with hepatitis B virus (HBV) integration.

    Commonly used for HCV/HBV infection studies and drug screening.

    Source: Japanese Collection of Research Bioresources (JCRB) Cell Bank (original) or the Liver Cancer Institute, Fudan University (as cited in your study).

#### 2.1.3. VX2 Cell Line

• Species: *Oryctolagus cuniculus* (rabbit).
• Sex: Not specified (historically derived from a male Shope papilloma virus‐induced tumor).
• Tissue origin: Squamous cell carcinoma (primary tumor‐induced by Shope papillomavirus).
• Official cell line name: VX2 (also written as VX‐2 or Vx2).
• RRID: CVCL_5J03 (Cellosaurus database).
• Key characteristics are as follows:

   Model applications:

    Widely used for in vivo liver tumor implantation (rabbit HCC models) and interventional oncology studies (e.g., ablation and embolization). Rapidly growing, highly vascularized tumors mimicking human HCC angiogenesis.

    Pathology: Poorly differentiated squamous cell carcinoma with epithelial morphology.

    Source: Originally from the Laboratory of Viral Oncology, National Cancer Institute (USA); in your study, obtained from the Liver Cancer Institute, Zhongshan Hospital, Fudan University (Shanghai, China).

#### 2.1.4. Limitations Statement

“The cell lines used in this study (HCCLM3, Huh7, and VX2) were not formally authenticated via short tandem repeat (STR) profiling or tested for mycoplasma contamination during the described experiments. While these lines were obtained from reputable sources (the Liver Cancer Institute, Zhongshan Hospital, and Fudan University for HCCLM3/Huh7; established VX2 stock for rabbit models), we acknowledge this as a methodological limitation that may affect reproducibility. Future studies will implement routine authentication (e.g., ICLAC‐recommended STR analysis) and mycoplasma screening (e.g., PCR or enzymatic assays) to comply with best practices.”

### 2.2. Transfection and Stable Cell Lines

Plasmid transfection was performed using Lipofectamine 2000 Reagent (Life Technology, Thermo Fisher Scientific, DE, USA). Plasmids (pcDNA3.1‐SERPINE1, pcDNA3.1‐VEGFA), SERPINE1 virus (Ubi‐MCS‐3FLAG‐CBh‐gcGFP‐IRES‐puromycin), and two siRNAs (siMETTL3: 5′‐GCUACCUGGACGUCAGUAUTT‐3′, siIGF2BP1: 5′‐TCTGCAACTCGTTCACCGT‐3′) were purchased from Genechem (Shanghai, China). An empty vector was used as a negative control. The transfection procedures strictly followed the manufacturer’s instructions for Lipofectamine 2000 Reagent (Invitrogen). For one group, a total of 5 × 10^5^ cells were seeded into a well of a six‐well plate. After transfection, quantitative real‐time PCR (qRT‐PCR) analysis or western blot analysis was used to verify the transfection efficiency.

### 2.3. RNA Extraction and qRT‐PCR

The detailed procedure of qRT‐PCR was the same as the methods described previously [32]. The expression level of each gene was normalized to that of ACTIN, which served as an internal control according to the 2^−ΔΔCt^ method. Primers (synthesized by Sunya, China) for human genes and rabbit genes can be found in the Supporting Information.

### 2.4. RNA‐Seq Assay

RNA sequence data of this study have been deposited at the SRA database under Accession Number: PRJNA792308; Our SRA records will be accessible with the following link after the indicated release date: https://www.ncbi.nlm.nih.gov/sra/PRJNA792308.

For the RNA‐Seq assay, after constructing libraries, sequencing data were operated with a GitHub package SOAPnuke (v1.5.2) (https://github.com) to remove the following elements: a. reads with sequencing adapter; b. low‐quality reads; c. reads whose “N” base ratio is more than 5%. Subsequently, clean reads were transformed to FASTQ format. HISAT2 (v2.0.4) (https://www.ccb.jhu.edu/software/hisat) was used to map the clean reads. And then, splicing genes was fuzed by EricScript (v0.5.5) (https://rnaseq-mats.sourceforge.net/) or rMATS (V3.2.5) (https://bowtiebio.sourceforge.io/). Afterwards, RSEM (v1.2.12) was used to calculate the expression level of gene (https://github.com/deweylab/RSEM). Finally, R (https://www.r-project.org/) was used to perform the analysis with *Q* value ≤ 0.05. GO (https://www.geneontology.org/) and KEGG (https://www.kegg.jp/) enrichment analysis of annotated different expression genes helped us to understand the change of the phenotype.

### 2.5. Western Blot

Cells were harvested at the indicated times. In each pole, 20 µg of total protein was used for electrophoresis. The membrane was blocked with 5% nonfat milk at room temperature for 1 h and then incubated with primary antibodies overnight at 4°C. Subsequently, a corresponding secondary antibody was applied to the membrane and incubated for 2 h at room temperature. The protein bands were visualized using a chemiluminescence ECL kit (Tanon, Shanghai, China). The antibodies used can be found in the Supporting Information.

### 2.6. GEPIA and HPA and STRING

The correlation of SERPINE1 expression with overall survival of HCC patients as well as HCC pathological stages was evaluated by GEPIA2 (https://gepia2.cancer-pku.cn) and the TCGA database (https://www.oncolnc.org/). The statistical method applied was the Kaplan–Meier method with a log‐rank test. The expression score of HCC and normal liver tissue in the HPA database described an estimated SERPINE1 level. The STRING database (https://string-db.org/cgi/input.pl) has been widely used to predict protein–protein interactions (PPI). In this study, we sought the possible interactive proteins with SERPINE1.

### 2.7. Ablation Model

New Zealand White rabbits were used to establish liver cancer in situ model. The rabbits were first planted with VX2 cells subcutaneously. After the xenografts grew to about 1 cm^3^, tumors were cut into small pieces (about 2–3 mm^3^) and were ready for transplantation. The rabbits were fasted for 12 h before the xenografts in situ transplantation. When the transplantation started, the rabbits were anesthetized with 2% pentobarbital sodium and the skin was cleaned. After the transplantation, a gelatin sponge was used to stop the bleeding, and 400,000 units of penicillin were used for three consecutive days. About 2 weeks after the establishment of the VX2 rabbit model, the rabbits were examined by ultrasound (MyLab Twice from Esaote, Italy), and the diameters of xenografts were 9.50 ± 1.56 mm (8–13 mm). The laser ablation system was Echo Laser X4 (the emitted laser wavelength was 1064 nm, and the fiber diameter was 300 μm). During ablation, the power was selected to be 4 W, and the output energy was about 100 J. We damaged 1/3–2/3 of the tumor to establish a residual tumor (partial ablation) model and created the condition of sublethal heat treatment. We used color Doppler ultrasound and shear wave viscoelasticity to examine the lesions at 3, 7, and 14 days after ablation, respectively.

### 2.8. Luciferase Reporter Assay

The luciferase reporter assay was performed according to the manufacturer’s instructions (11402ES60, Yeasen). HCCLM3 and Huh7 cells were seeded in six‐well plates and transfected with the wide‐type SERPINE1‐responsive luciferase reporter construct (SERPINE1‐WT), the mutant SERPINE1 (SERPINE1‐MUT)‐responsive luciferase reporter construct (SERPINE1‐MUT), the wide‐type METTL3 plasmids, or the IGF2BP1, accordingly. At 24 h posttransfection, cell lysates were incubated with 10 μg/mL of firefly and TK separately for 10 min and luciferase activities were measured using a Dual‐Luciferase Reporter Assay System (Promega, Madison, WI, USA) and a microplate luminometer (Promega). The firefly luciferase activities were corrected by the corresponding *renilla* luciferase activities. Results were representative of three independent experiments.

### 2.9. ELISA

After the cells were transfected, the cell culture supernatant was collected. The human SERPINE1 kit (ab269373, abcam) and VEGF‐A Human ELISA Kit (BMS277‐2, Thermo Fisher, Invitrogen) were used to analyze the SERPINE1 and VEGFA concentrations following the manufacturer’s instructions.

### 2.10. RNA Decay Assay

RNA decay assay was performed following the study of Qing et al. [33]. In brief, HCCLM3 and Huh7 cells were seeded in 6‐cm plates at 50% confluency. After 24 h, each 6‐cm plate was re‐seeded into three 6‐cm plates. After 48 h, actinomycin D was added to 3 mg/mL at 6 , 3 , and 0 h before trypsinization and collection. The total RNA was purified by a DNase‐I digestion step on column. RNA quantities were determined by RT‐qPCR. The mRNA decay rate was calculated by the equations as described by Qing et al. [33].

### 2.11. Subcutaneous Xenograft in Nude Mice

In order to mimic the sublethal heat‐treatment subcutaneous implantation model, we prepared the cells first. HCCLM3 cells were heated at 46°C for 10 min and seeded into a 6‐cm dish to mimic the sublethal status, then the dishes were changed medium twice a day as described previously [26]. About 2 days later, 5 × 10^5^ stable sublethal heat‐treated HCCLM3 cells were injected subcutaneously into BALB/C nude mice. About 7 days after implantation, the control group was given PBS 0.1 mL every other day, while the group of PAI‐039 was given 1 mg/kg tiplaxtinin (PAI‐039) intraperitoneal injection every other day. About 25 days later, mice were sacrificed.

### 2.12. Organoid

HCC PDO organoids were established according to a previous study, and 3 PDOs were adopted in this study [34]. For the heated group, PDOs were collected after centrifugation at 300 g for 10 min and were then incubated at 46°C for 10 min. PAI‐1 inhibitor PAI‐039 (final concentration: 10 μmol/L) was added to the PDO culture medium for 4–5 days. Then the heated PDOs proceeded to passage, and organoid pictures were taken every 2–3 days before formalin fixation. PDOs were pre‐embedded with 1% agarose (Biowest agarose, 111860) and fixed with 4% phosphate‐buffered formalin, embedded in paraffin, and cut at 4 μm for hematoxylin‐eosin and immunofluorescence analysis with standard procedure [35]. Immunofluorescence staining was performed as described previously [36]. Antibodies used can be found in the Supporting Information. Images were obtained with a confocal microscope (Olympus).

### 2.13. Human HCC Sample

A total of 5 HCC patients received biopsy in the Department of Interventional Radiology during 2021 and were diagnosed with HCC. All patients provided written informed consent in this study. The ethical approval can be found in the Animal and Human Ethics Files.

### 2.14. Tube Formation Assay

The formation of tubes by HUVEC cells on Matrigel was performed as previously described [37]. Briefly, supernatant from HCCLM3 or Huh7 cells with different transfection conditions was put into the HUVEC cells. Nine hours later, the tube formation was detected by photography.

### 2.15. Immunohistochemistry

Samples were dehydrated by ethanol at different concentrations and finally embedded in paraffin wax. Paraffin sections were made into pieces and dried overnight at 60°C. Serial paraffin sections were rehydrated at decreasing concentrations of ethanol after being deparaffinized. The SABC kit was used according to the manufacturer instructions (BOSTER, Wuhan, China). Antibodies used can be found in the Supporting Information. Finally, the sections were imaged by a phase‐contrast microscope (Canon, Japan).

### 2.16. Statistical Analysis

All of the analyses in this study were performed in GraphPad Prism 8.00 (GraphPad Software, San Diego, USA). Student’s *t*‐test was used for statistical analysis. A *p*‐value < 0.05 was considered statistically significant. Data are presented as the mean ± SEM.

## 3. Results

### 3.1. Sublethal Heat Ablation Treatment Stimulates SERPINE1 Expression In Vitro and In Vivo

In our previous work, we successfully established the model of sublethal heat treatment [26]. Therefore, we used this model to perform next generation sequencing, and got the top 25 increased mRNA and top 25 decreased mRNA as the heat map showed (Figure 1A).

Figure 1Sublethal heat ablation treatment stimulates SERPINE1 expression in vitro and in vivo. (A) The heat map of “top 25 increased genes” and “top 25 decreased genes.” (B) The Venn diagram was drawn based on three GO terms “angiogenesis,” “positive regulation of angiogenesis,” and “extracellular exosome.” (C) The concrete “cross genes” of the Venn diagram. (D, E) The relative SERPINE1 mRNA and protein level detected by qRT‐PCR and western blot, respectively. (F) The TCGA dataset showed the relationship between SERPINE1 expression and the overall survival rate of HCC. (G) The level of SERPINE1 in different stages of HCC. (H) The representative image showed the SERPINE1 expression in HCC versus normal tissues in the HPA database. (I) The expression of SERPINE1 in HCC and paracancer tissues from the HCC patients. (J) Schematic diagram of the establishment of the VX2 rabbit ablation model. (K) Successful establishment of VX2 liver cancer in situ. The representative tumor was 7 × 7 mm^2^. a: tumor; b: normal liver tissue. (L) After the anesthesia, two doctors gave the microwave ablation to the rabbit models. During ablation, the power is selected to be 4 W, and the output energy is about 100 J. (M‐O) The ultrasound image illustrated the general condition at 1, 3, and 7 days after microwave ablation. Red part: residual tumor; blue part: the ablated tissue; yellow part: normal liver tissue. (P) The ultrasound image and contrast‐enhanced ultrasonography illustrated the general condition. a: residual tumor; b: the ablated tissue; c: periablation inflammatory part; d: normal liver tissue (Q) The ultrasound image illustrated the general condition 14 days after microwave ablation. Red part: residual tumor; blue part: the ablated tissue; yellow part: normal liver tissue. (R) The gross picture showed residual tumor and normal liver surrounding the ablation part. a: tumor; b: the ablated tissue; c: periablation inflammatory part; d: normal liver tissue. (S) The H.E. staining (×10) showed the residual tumor and normal liver surrounding the ablation part. a: tumor; b: the ablated tissue; c: periablation inflammatory part; d: normal liver tissue. (T) The relative mRNA levels of SERPINE1 in residual rabbit liver cancer at different time points. (U) The relative protein levels of SERPINE1 of residual rabbit liver cancer at different time points. “NS,” not significant,  ^∗^ *p* < 0.05,  ^∗∗^ *p* < 0.01,  ^∗∗∗^ *p* < 0.001, and  ^∗∗∗∗^ *p* < 0.0001.

**Figure:**
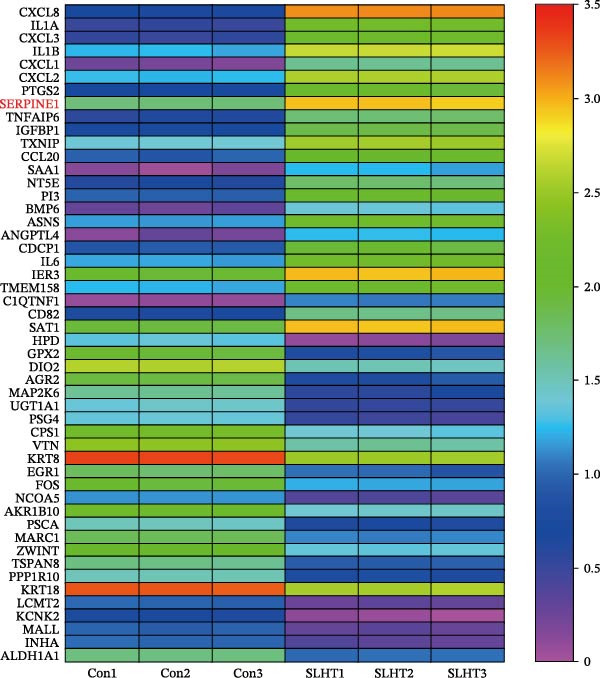
(A)

**Figure:**
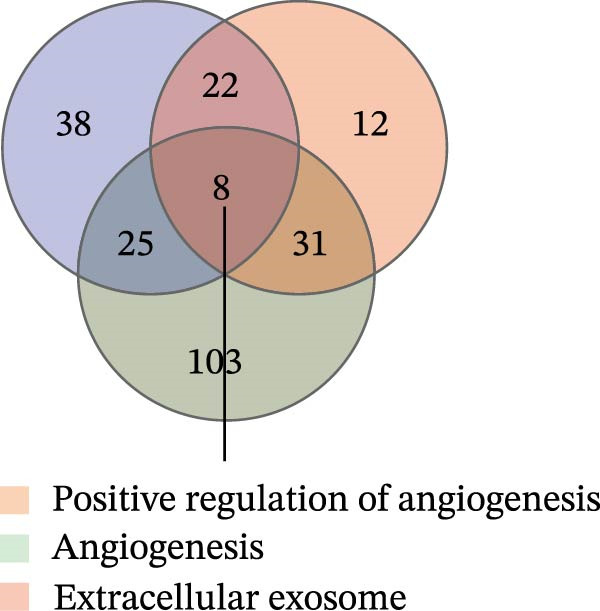
(B)

**Figure:**
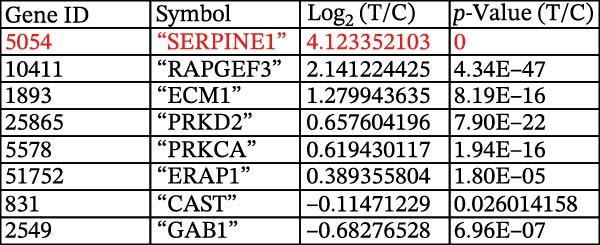
(C)

**Figure:**
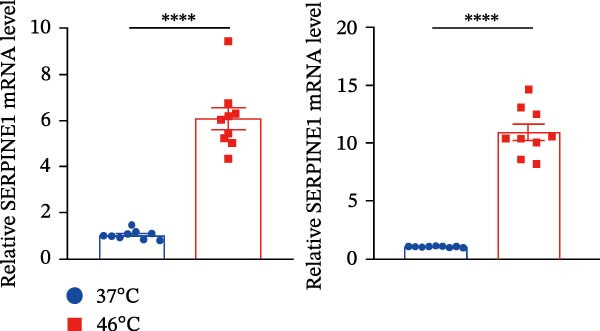
(D)

**Figure:**
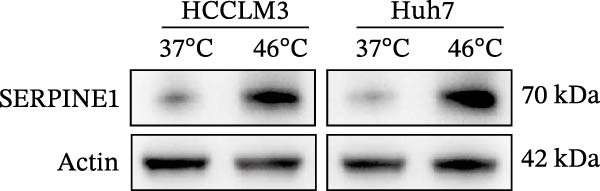
(E)

**Figure:**
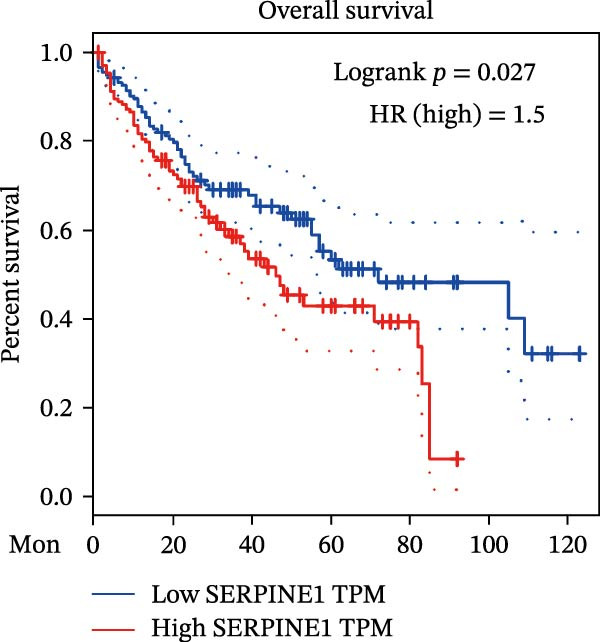
(F)

**Figure:**
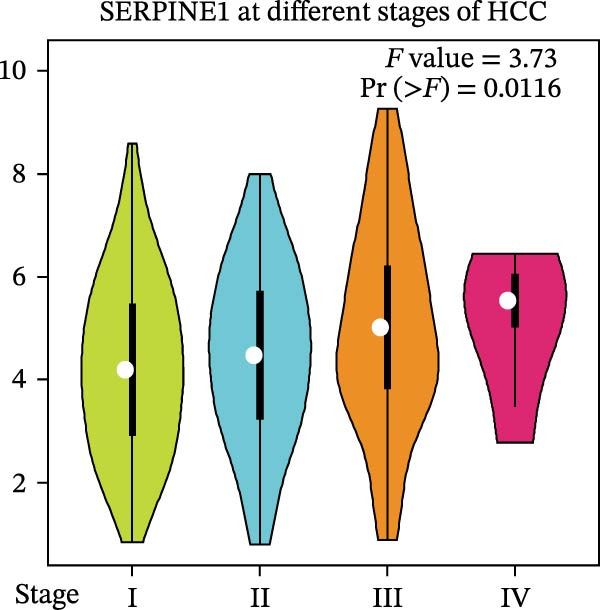
(G)

**Figure:**
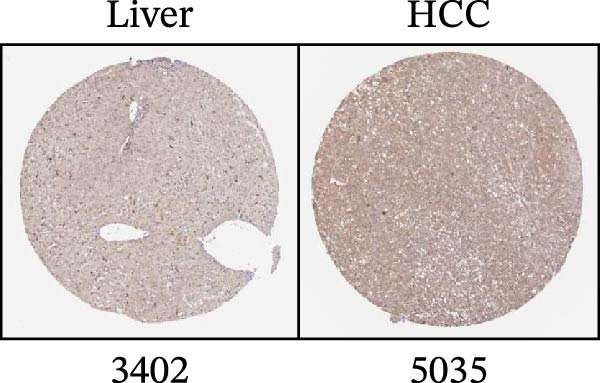
(H)

**Figure:**
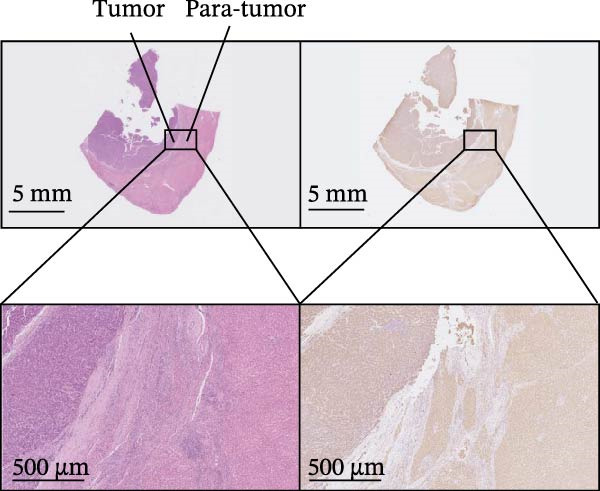
(I)

**Figure:**
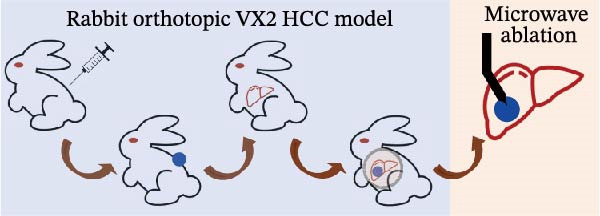
(J)

**Figure:**
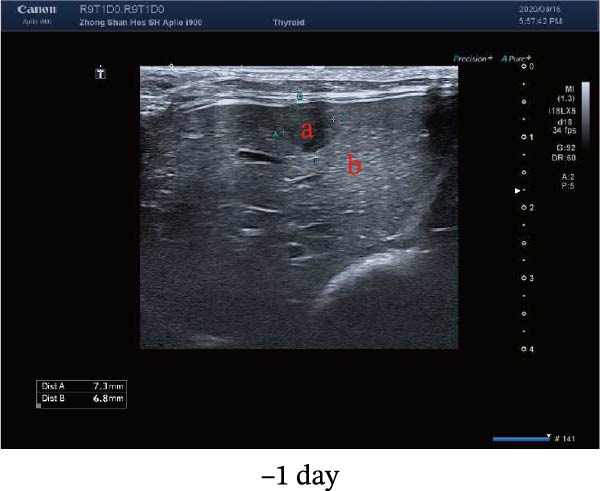
(K)

**Figure:**
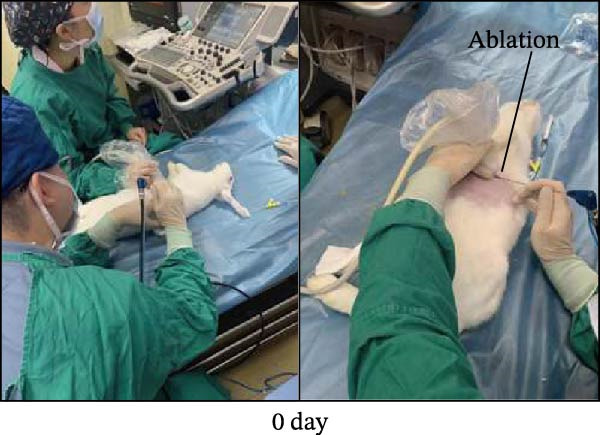
(L)

**Figure:**
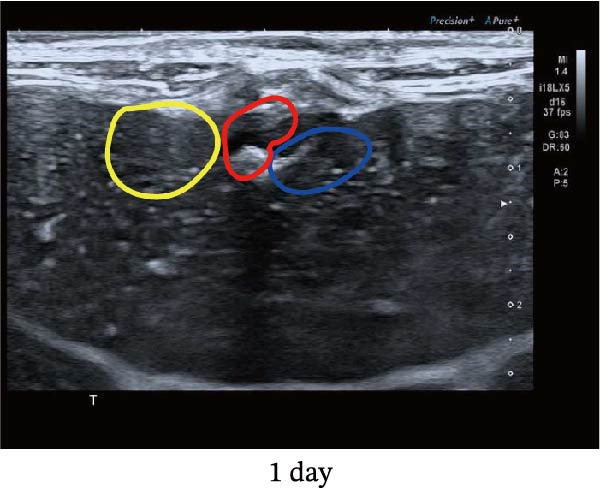
(M)

**Figure:**
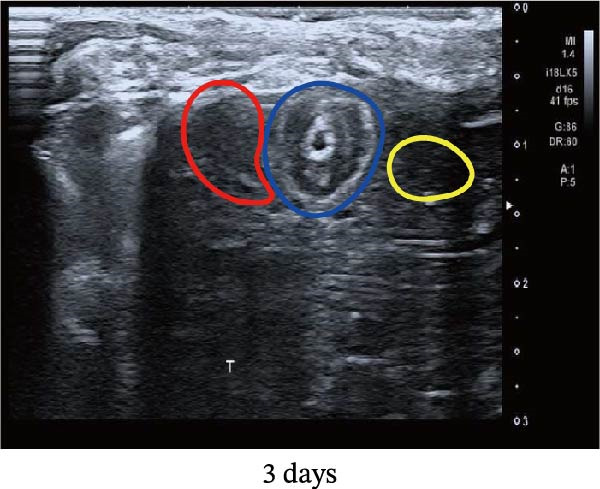
(N)

**Figure:**
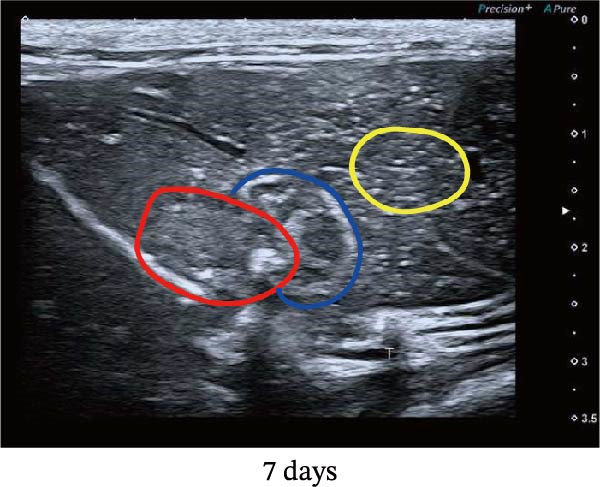
(O)

**Figure:**
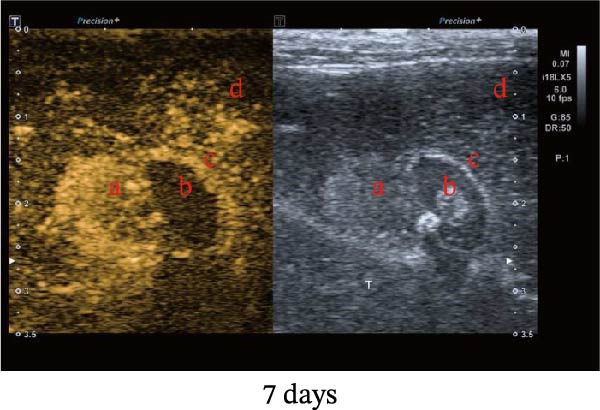
(P)

**Figure:**
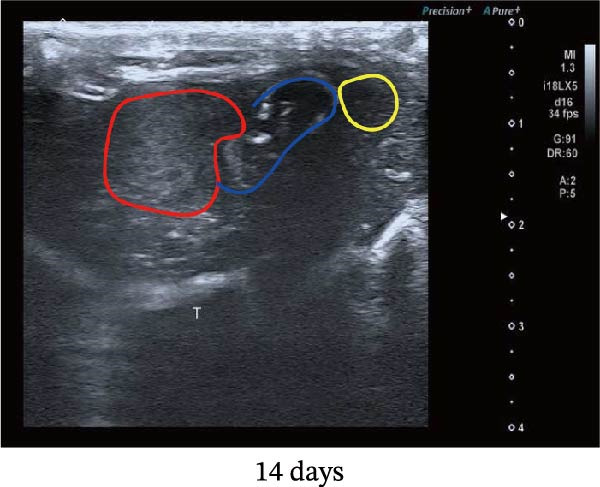
(Q)

**Figure:**
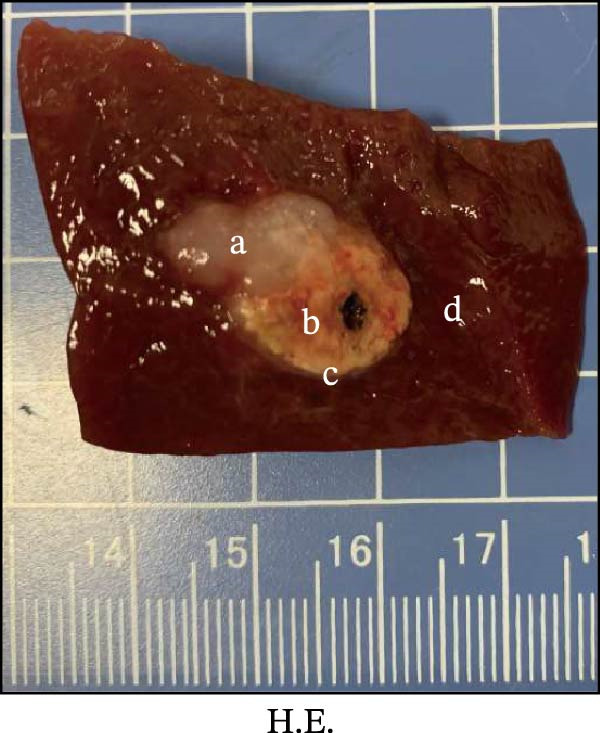
(R)

**Figure:**
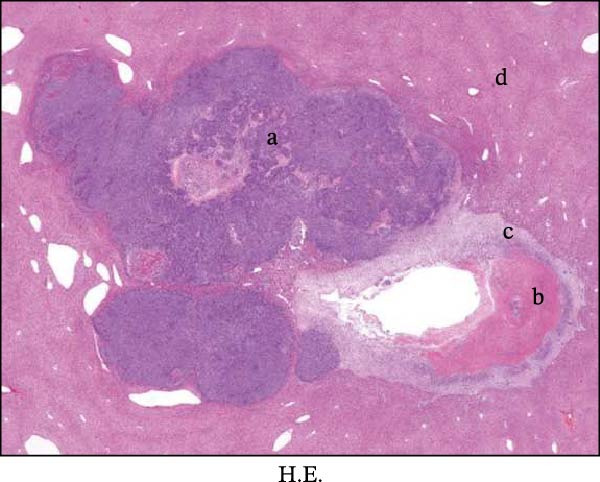
(S)

**Figure:**
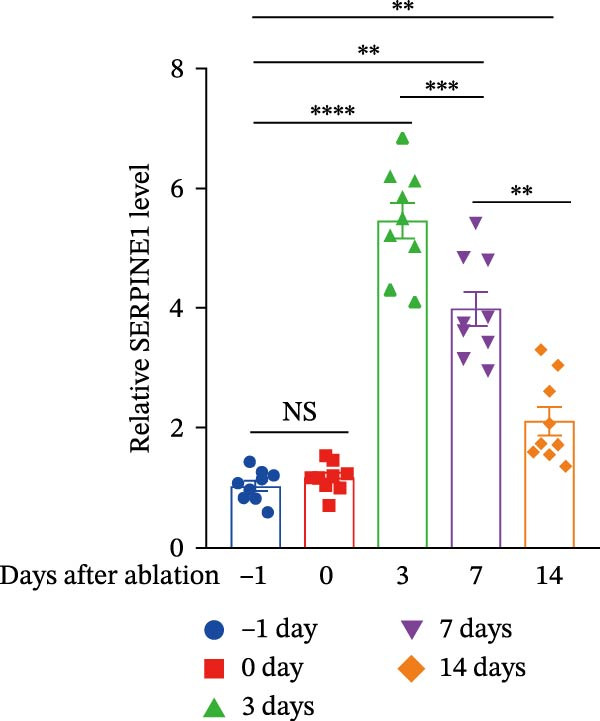
(T)

**Figure:**
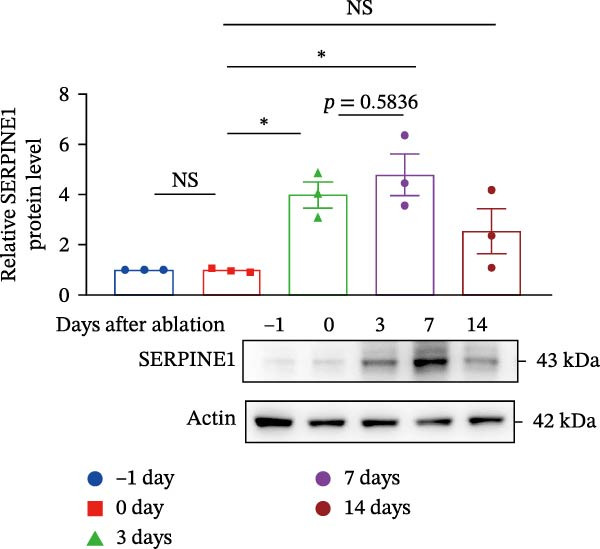
(U)

Tumor blood vessels were critical to the tumor growth, so we checked the crossover between GO terms “positive angiogenesis,” “angiogenesis,” and “extracellular exosome.” Consequently, the Venn diagram illustrated eight “intersection genes” which were “SERPINE1, RAPGEF3, ECM1, PRKD2, PRKCA, ERAP1, CAST, and GAB1” (Figure 1B).

Among them, “SERPINE1” obtained the largest alteration, which increased at least 16‐fold in the group of sublethal heat treatment compared with that in the control group (Figure 1C). This indicated that SERPINE1 might be involved in angiogenesis after sublethal heat treatment in HCC.

Furthermore, both qRT‐PCR and western blot analysis confirmed that sublethal heat treatment increased the SERPINE1 expression (Figure 1D).

To gain insight into the function of SERPINE1 in HCC, we first analyzed the expression of SERPINE1 and overall survival rate of HCC patients in the TCGA database. The LIHC datasets showed that the group with high expression of SERPINE1 had the lower survival time (Figure 1F). On the other hand, patients with higher tumor stages exhibited higher expression of SERPINE1 (Figure 1G). The HPA datasets illustrated that patients suffering from HCC had higher expression of SERPINE1 than the normal counterpart (Figure 1H), which was consistent with our cases (Figure 1I).

In order to mimic HCC with sublethal heat treatment, we established the ablated rabbit HCC model with VX2 (Figure 1J). We used ultrasound to precisely target the xenografts and control the ablation area, and left the residual HCC growing (Figure 1K–Q). The residual HCC tissue survived (Figure 1R,S). Compared with the group without ablation, SERPINE1 increased at 3 and 7 days after ablation. Later, SERPINE1 decreased gradually after ablation (Figure 1T,U).

### 3.2. SERPINE1/VEGFA Axis Contributes to Sublethal Heat Treatment‐induced Angiogenesis in HCC Cells

To study the concrete mechanism of SERPINE1, we first blocked the endogenous expression of SERPINE1 by either shRNA transfection or using the SERPINE1 inhibitor PAI‐039; however, sublethal heat treatment cannot elevate SERPINE1 upon PAI‐039 (Figure 2A–D). Notably, inhibition of SERPINE1 dramatically reduced the excretion of SERPINE1 in the supernatant, which could be partially rescued by sublethal heat treatment in HCCLM3 cells and Huhh7 cells (Figure 2E,F). Tube formation test showed that after being cultured with the supernatant from the cells that inhibited SERPINE1 expression either by shRNA or by PAI‐039, HUVEC cells exhibited a significant reduction in the tube formation ability (Figure 2G,H).

Figure 2SERPINE1/VEGFA axis contributes to sublethal heat treatment‐induced angiogenesis in HCC cells. (A) The mRNA level of SERPINE1 when knocked down at 37 or 46°C. (B) The protein level of SERPINE1 when knocked down at 37 or 46°C. (C) The mRNA level of SERPINE1 by the treatment of SERPINE1 inhibitor PAI‐039 at 37 or 46°C. (D) The protein level of SERPINE1 by the treatment of SERPINE1 inhibitor PAI‐039 at 37 or 46°C. (E) ELISA showed the extracellular levels of SERPINE1 in the supernatant when SERPINE1 was knocked down at 37 or 46°C. (F) ELISA showed the extracellular levels of SERPINE1 in the supernatant when SERPINE1 was knocked down at 37 or 46°C. (G) The tube formation assay of HUVECs. Knockdown of SERPINE1 was performed in HCCLM3 and Huh7 cells. The supernatant from HCCLM3 or Huh7 with the indicated treatment was collected and used to culture HUVEC for 48 h. (H) Relative number of meshes of the tube formation assay. (I) The tube formation assay of HUVECs. The supernatant from HCCLM3 or Huh7 treated with or without PAI‐039 was collected and used to culture HUVEC for 48 h. (J) Relative number of meshes of the tube formation assay. (K) STRING analysis showed the potential correlated proteins with SERPINE1. (L) The correlation between SERPINE1 and VEGFA. (M) The correlation between SERPINE1 and SMAD2. (N) The correlation between SERPINE1 and SMAD3. (O) The correlation between SERPINE1 and SMAD7. (P) The protein level and its relative quantification of VEGFA when SERPINE1 was knocked down in HCCLM3 and Huh7 at 37 or 46°C. (Q) The relative VEGFA expression in the supernatant from HCCLM3 or Huh7 when SERPINE1 was inhibited at 37 or 46°C. (R) The SERPINE1 and VEGFA protein expression in HCCLM3 and Huh7 cells in which SERPINE1 was knocked down and treated with or without additional VEGFA. (S) The tube formation assay of HUVECs. The supernatant from HCCLM3 or Huh7 was collected and used to culture HUVEC for 48 h. (T) Relative number of meshes of the tube formation assay. “NS,” not significant,  ^∗^ *p*  < 0.05,  ^∗∗^ *p*  < 0.01,  ^∗∗∗^ *p*  < 0.001, and  ^∗∗∗∗^ *p*  < 0.0001.

**Figure:**
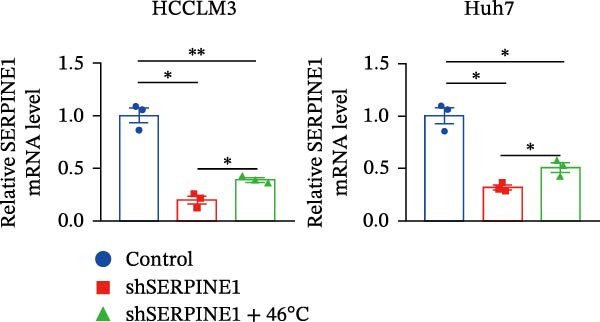
(A)

**Figure:**
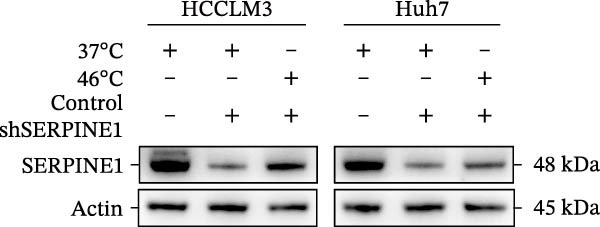
(B)

**Figure:**
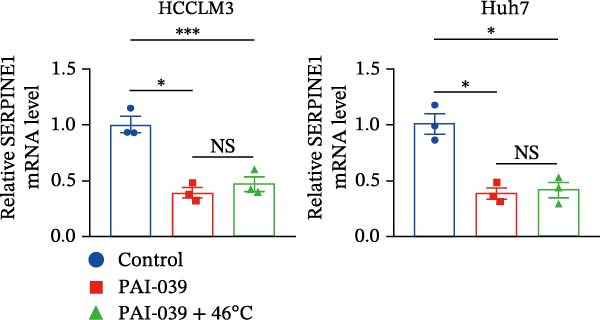
(C)

**Figure:**
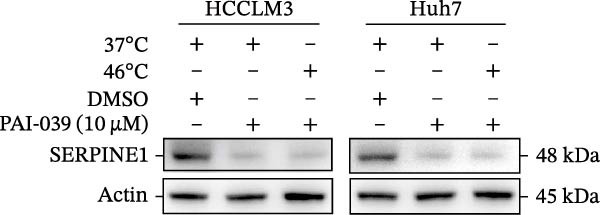
(D)

**Figure:**
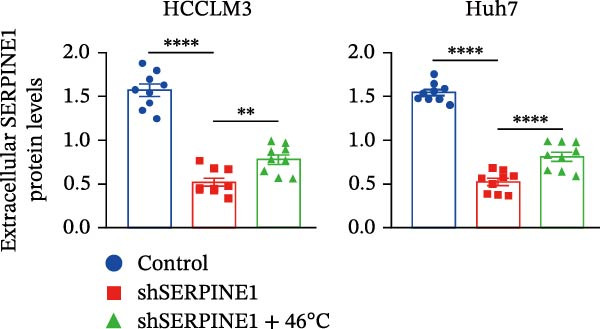
(E)

**Figure:**
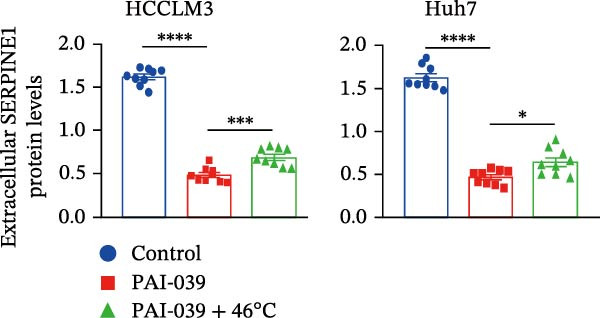
(F)

**Figure:**
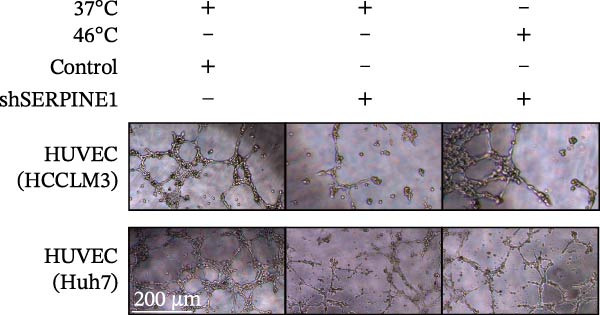
(G)

**Figure:**
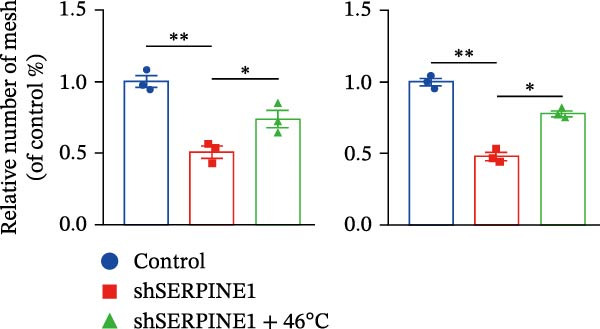
(H)

**Figure:**
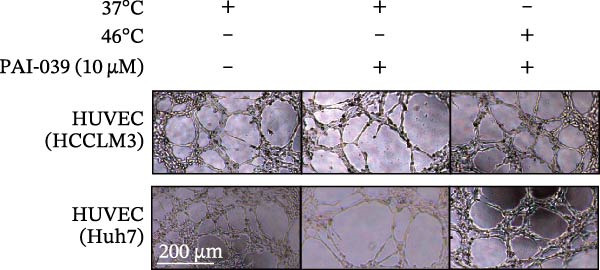
(I)

**Figure:**
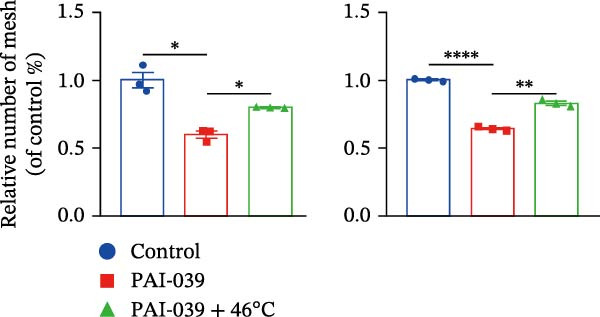
(J)

**Figure:**
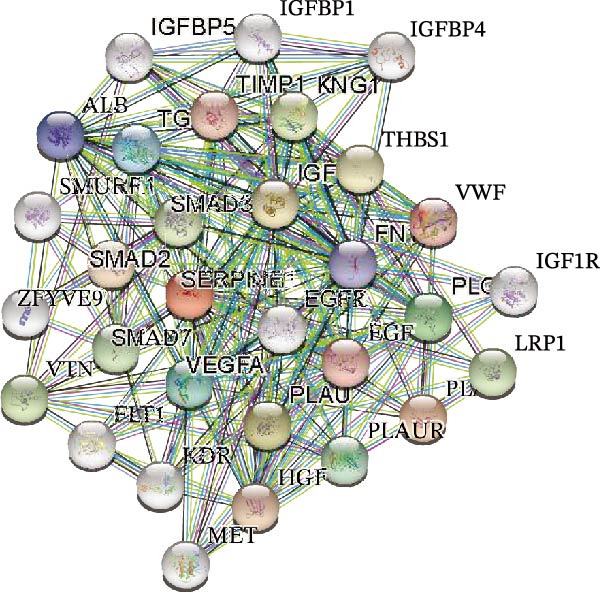
(K)

**Figure:**
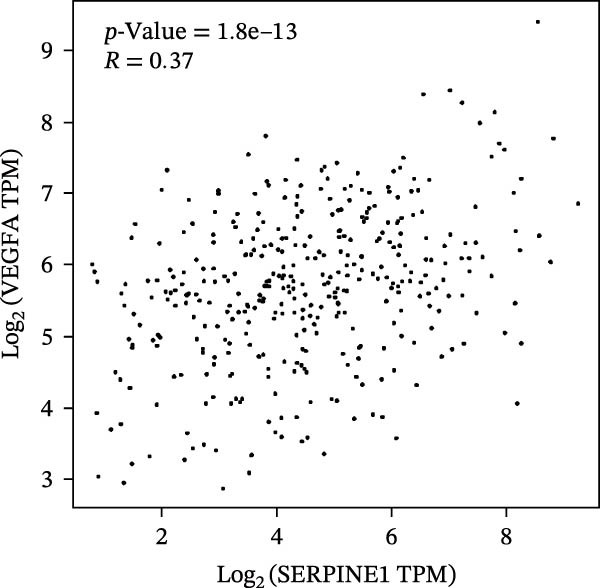
(L)

**Figure:**
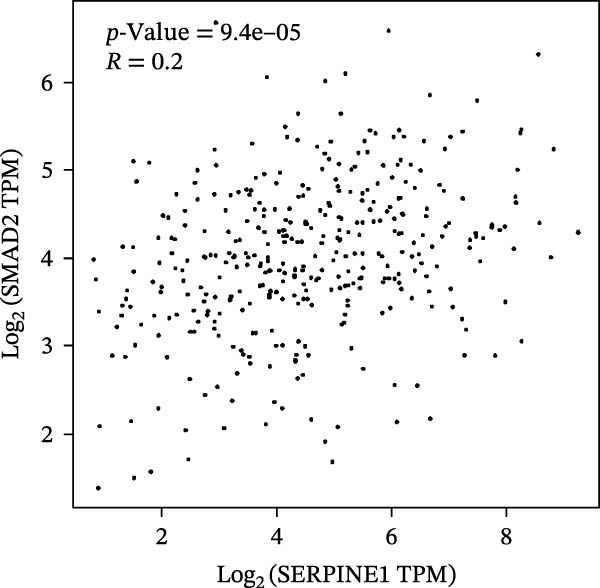
(M)

**Figure:**
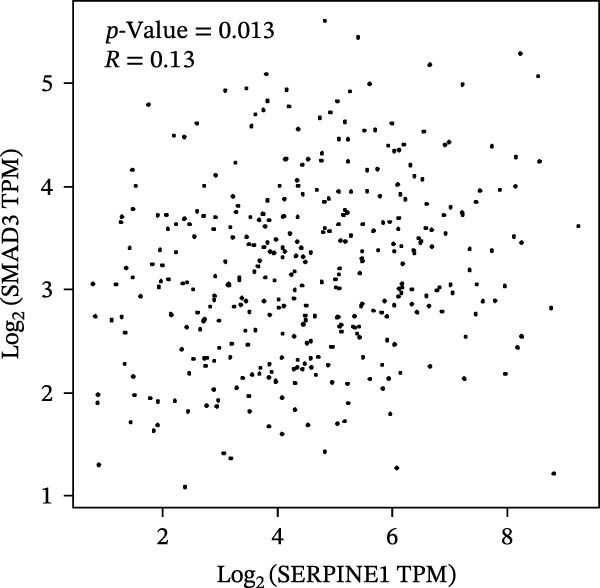
(N)

**Figure:**
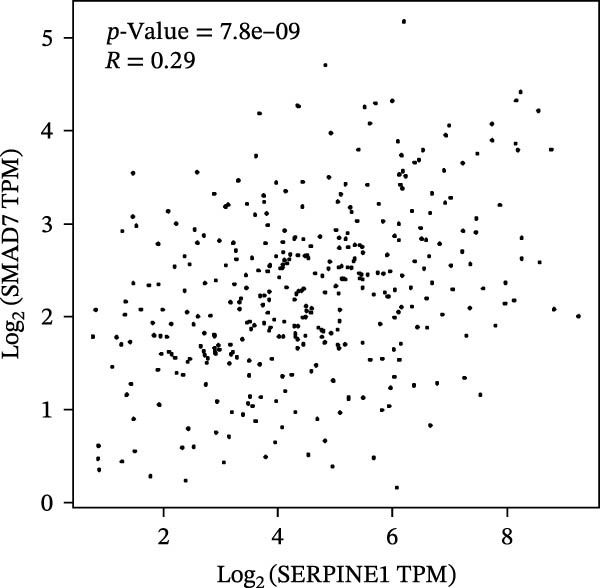
(O)

**Figure:**
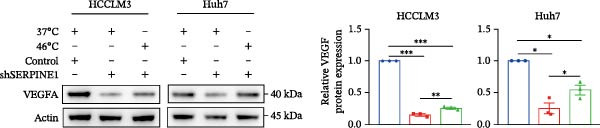
(P)

**Figure:**
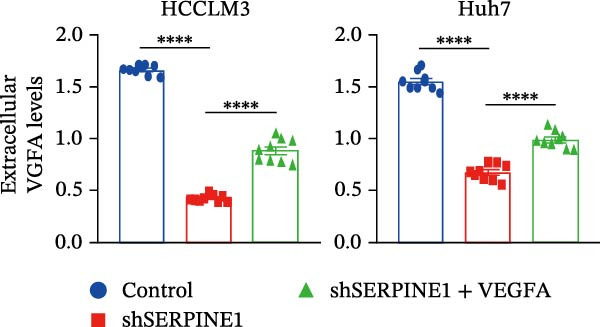
(Q)

**Figure:**
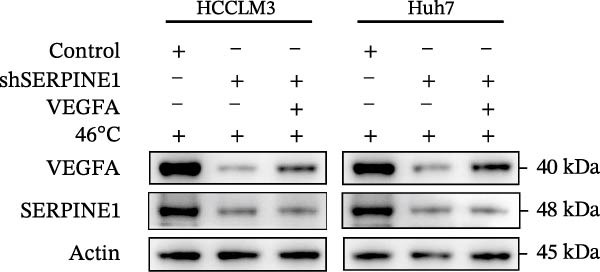
(R)

**Figure:**
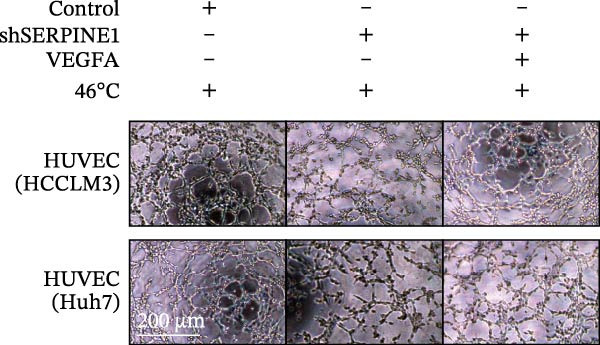
(S)

**Figure:**
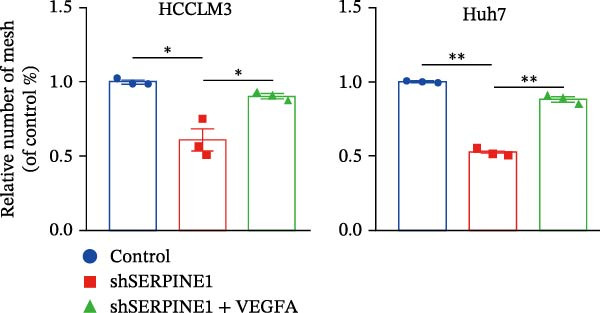
(T)

To further explore the downstream effector that mediates the SERPINE1‐induced angiogenesis, we analyzed the PPI by STRING (https://string-db.org). Interestingly, some members of the SMAD family and VEGFA were shown to be closely interacting with SERPINE1 (Figure 2K). Furthermore, in the TCGA LIHC dataset, SMAD2, SMAD3, SMAD7 and VEGFA were significantly correlated with SERPINE1 (Figure 2L–O). Among them, the *R* value of VEGFA was up to 0.37, which strongly indicated a positive correlation between SERPINE1 and VEGFA.

As expected, we did observed that SERPINE1 reduction decreased VEGFA expression in HCCLM3 and Huh7 cells as well as the secreted VEGFA in the supernatant (Figure 2P,Q). On the other hand, addition of VEGFA could facilitate the tube formation that was reduced by SERPINE1 inhibition (Figure 2R–T).

### 3.3. Sublethal Heat Treatment‐induced SERPINE1 Expression in a METTL3/m6A/IGF2BP1‐dependent Manner

In our previous study, we found that METTL3 promoted invasion in HCC after sublethal heat treatment [26]. Angiogenesis is positively related to invasion and metastasis in HCC [11]. Therefore, we proposed that METTL3 could regulate SERPINE1 in angiogenesis. Surprisingly, inhibition of METTL3 reduced the expression of SERPINE1 both in mRNA and protein levels, and manipulation of SERPINE1 had no effect on METTL3 expression (Figure 3A–D). The data above reminded us that METTL3 might be upstream of SERPINE1.

Figure 3Sublethal heat treatment‐induced SERPINE1 expression in a METTL3/m6A/IGF2BP1‐dependent manner. (A) Relative SERPINE1 mRNA level when METTL3 was knocked down with/without plus SERPINE1 in HCCLM3 or Huh7. (B) Relative METTL3 mRNA level when METTL3 was knocked down with/without plus SERPINE1 in HCCLM3 or Huh7. (C) Relative METTL3 and SERPINE1 protein levels when METTL3 was knocked down with/without plus SERPINE1 in HCCLM3 or Huh7. (D) Relative METTL3 and SERPINE1 mRNA levels when SERPINE1 was knocked down in HCCLM3 or Huh7. (E) Relative METTL3 and SERPINE1 protein levels when SERPINE1 was knocked down in HCCLM3 or Huh7. (F) The schematic of predicted m6A modification sites of SERPINE1 from RMBase. (G) Wild‐type or m6A consensus sequence mutant SERPINE1 cDNA was fuzed with a firefly luciferase reporter. Mutation of m6A consensus sequences of SERPINE1 relieved the posttranscriptional repression of SERPINE1 with METTL3 in HCCLM3 and Huh7. (H) The tube formation assay of HUVECs. The supernatant from HCCLM3 or Huh7 was collected and used to culture HUVEC for 48 h. The condition was that METTL3 was knocked down with/without plus SERPINE1 in 46°C. (I) Relative number of meshes of the tube formation assay of Figure 3H. (J) RNA stability assay of the condition where METTL3 was knocked down with/without plus SERPINE1 at 46°C. (K) Relative IGF2BP1 and SERPINE1 mRNA level when IGF2BP1 was knocked down in HCCLM3 or Huh7. (L) Relative IGF2BP1 and SERPINE1 protein level when IGF2BP1 was knocked down in HCCLM3 or Huh7. (M) Relative IGF2BP1 and SERPINE1 mRNA level when SERPINE1 was knocked down in HCCLM3 or Huh7. (N) Relative IGF2BP1 and SERPINE1 protein level when SERPINE1 was knocked down in HCCLM3 or Huh7. (O) Wild‐type or m6A consensus sequence mutant SERPINE1 cDNA was fuzed with a firefly luciferase reporter. Mutation of m6A consensus sequences of SERPINE1 relieved the posttranscriptional repression of SERPINE1 with IGF2BP1 in HCCLM3 and Huh7. “NS,” not significant,  ^∗^ *p*  < 0.05,  ^∗∗^ *p*  < 0.01,  ^∗∗∗^ *p*  < 0.001, and  ^∗∗∗∗^ *p*  < 0.0001.

**Figure:**
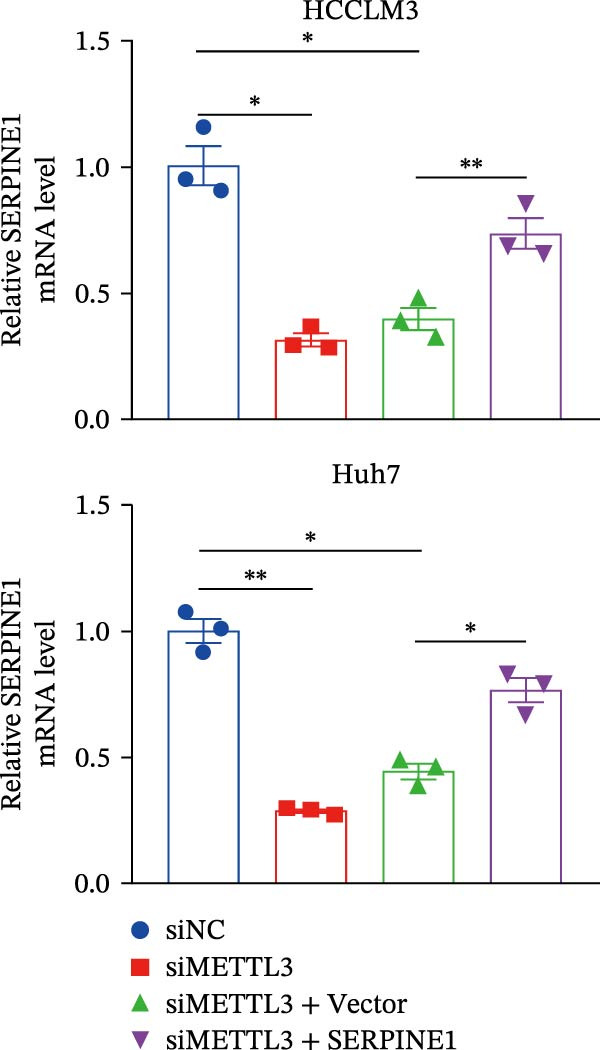
(A)

**Figure:**
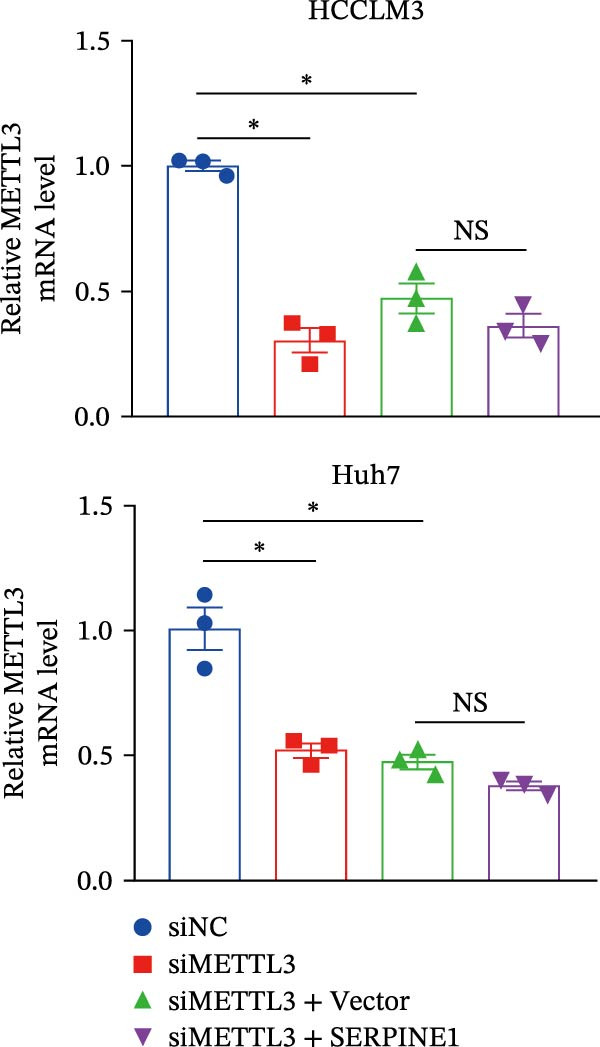
(B)

**Figure:**
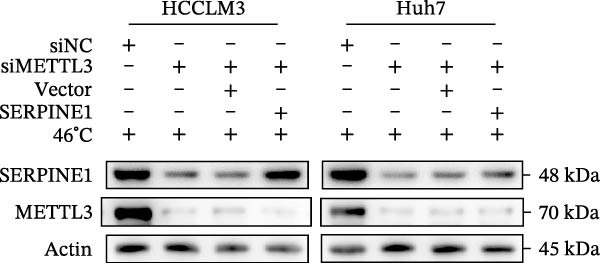
(C)

**Figure:**
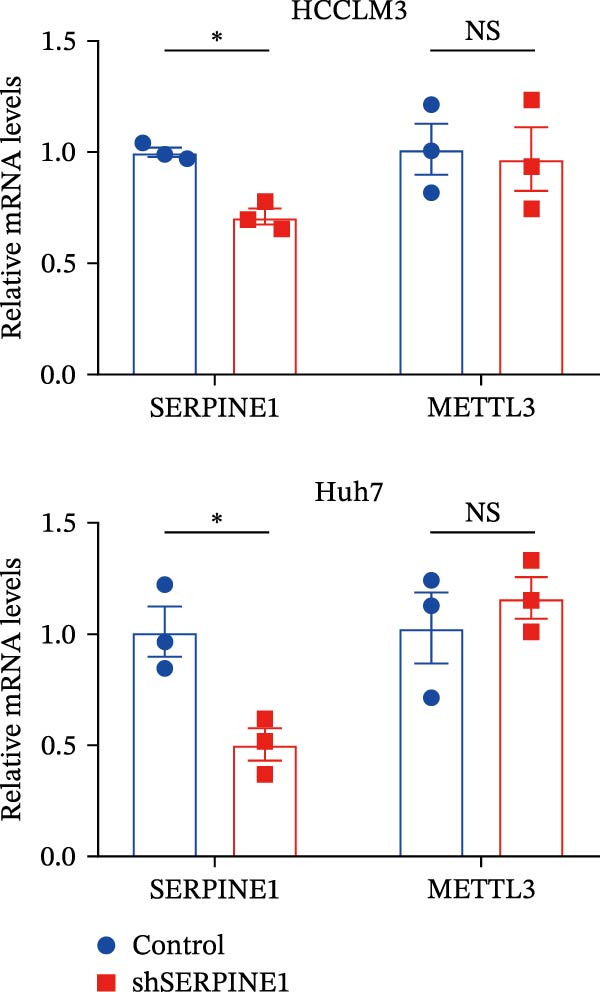
(D)

**Figure:**
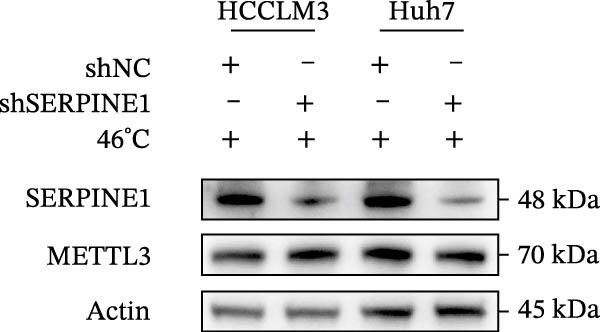
(E)

**Figure:**
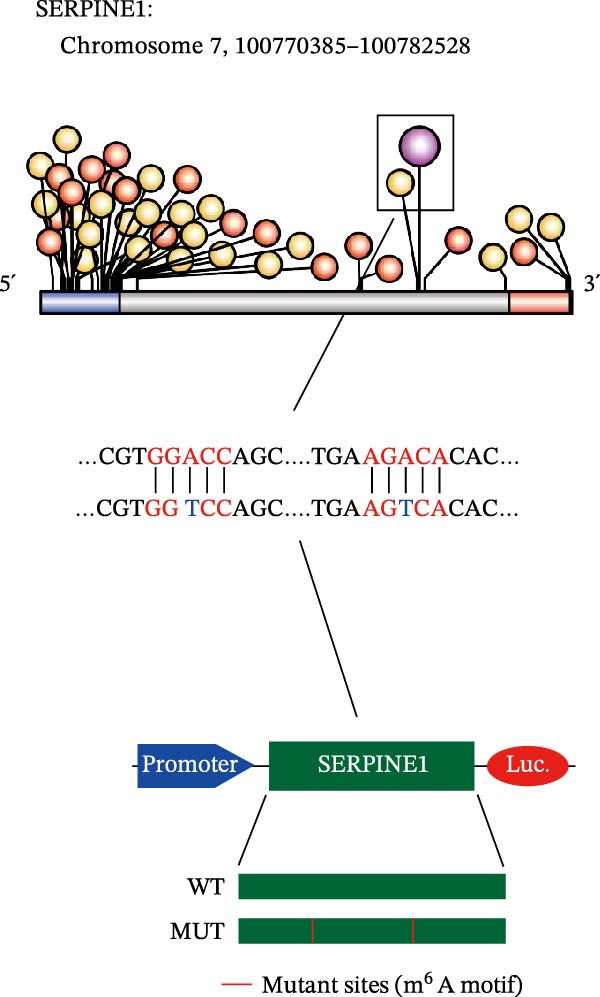
(F)

**Figure:**
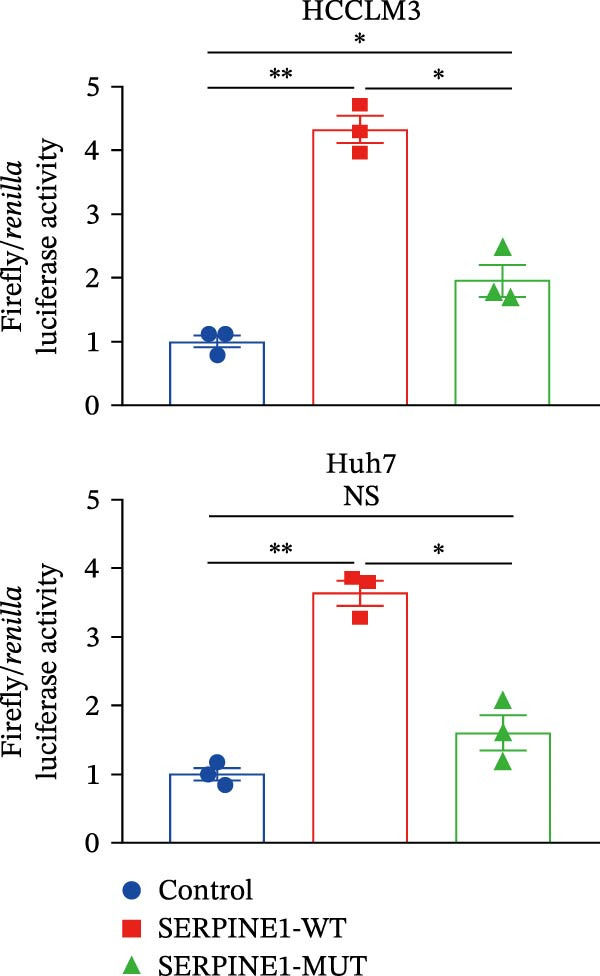
(G)

**Figure:**
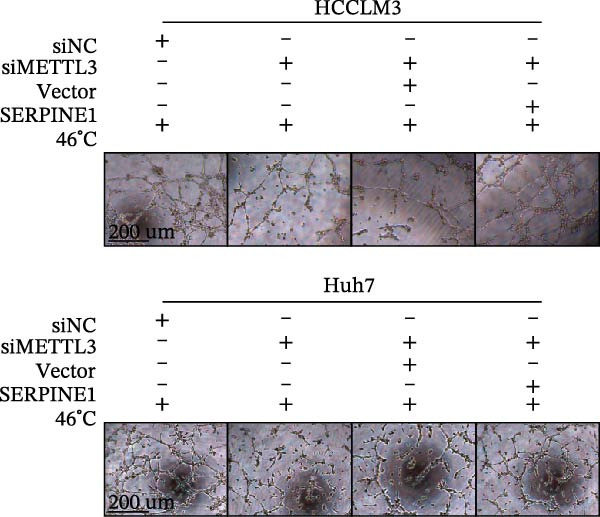
(H)

**Figure:**
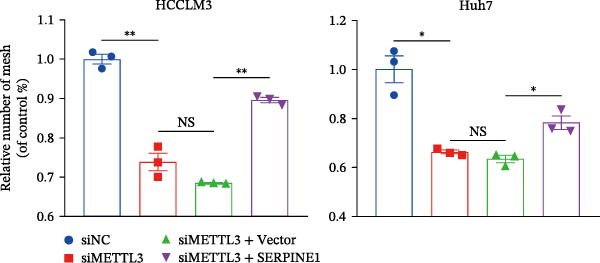
(I)

**Figure:**
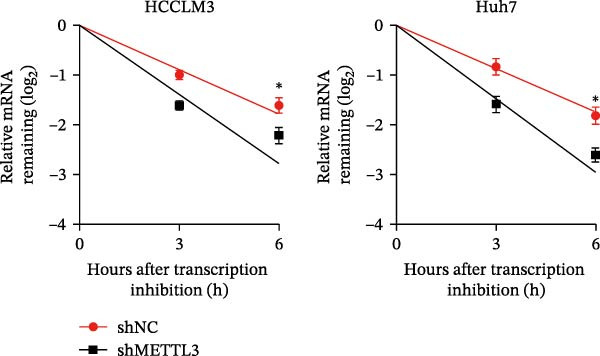
(J)

**Figure:**
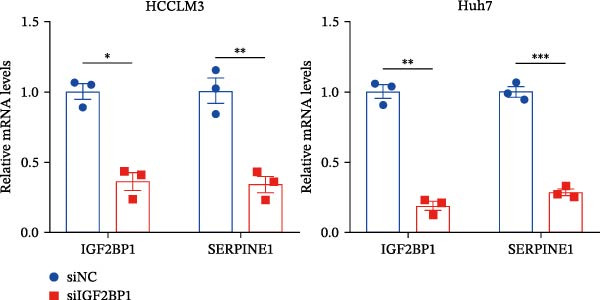
(K)

**Figure:**
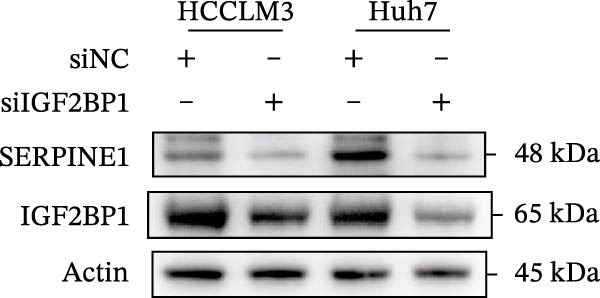
(L)

**Figure:**
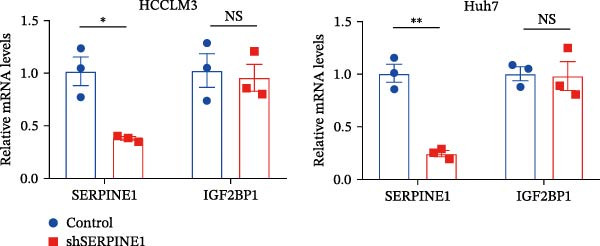
(M)

**Figure:**
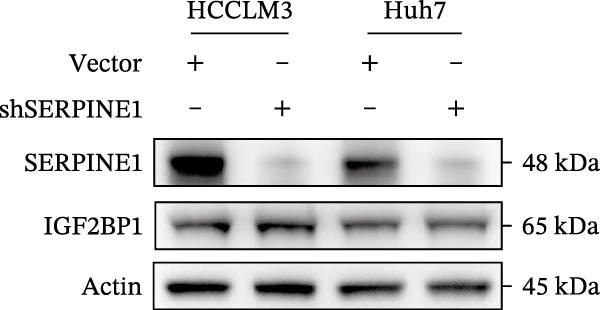
(N)

**Figure:**
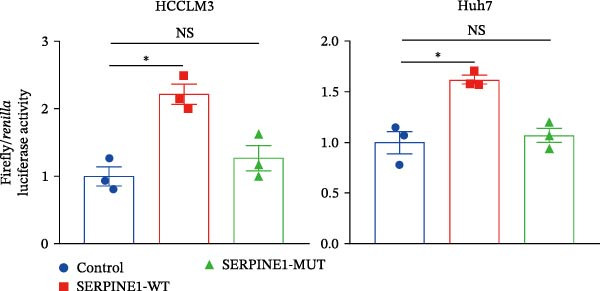
(O)

Since METTL3 is a methyltransferase enzyme, we wonder if SERPINE1 is regulated by METTL3 in an m6A manner. We analyzed the m6A modification site of SERPINE1 mRNA on RMBase 2.0 (https://rna.sysu.edu.cn/rmbase). Consequently, there were over 40 predicted m6A sites (Figure 3F). According to the prediction, we constructed SERPINE1‐MUT plasmid by choosing two m6A modification sites with the highest motif score (the purple one) and its neighborhood. As a result, the luciferase assay showed that the METTL3 significantly increased the firefly/*renilla* activity of wide‐type SERPINE1 but had little effect on the SERPINE1‐MUT group (Figure 3G). What is more, silence of METTL3 dramatically inhibited the tube formation ability, which could be blocked by overexpression of SERPINE1 (Figure 3H,I).

Next, we detected the half‐time of SERPINE1 mRNA and found that METTL3 knockdown decreased SERPINE1mRNA stability in HCCLM3 and Huh7 cells, respectively (Figure 3J). It is well established that IGF2BP1 is a m6A “READER” that can recognize m6A modification and retards the mRNA decay [38]. Consequently, inhibition of IGF2BP1 decreased SERPINE1 mRNA stability (Figure 3K,L), but manipulation of IGF2BP1 did not alter SERPINE1 expression (Figure 3M,N). The luciferase reporter showed that IGF2BP1 significantly stimulated the firefly/*renilla* activity of wide‐type SERPINE1 but had little effect on the SERPINE1‐MUT group (Figure 3O).

### 3.4. SERPINE1 Is Upregulated in HCC Organoid Model Under Sublethal Heat Treatment

Next, we tested the effect of sublethal heat treatment on SERPINE1 expression in human HCC PDO models. Consistent with our previous findings, after sublethal heat (46°C) treatment for 10 min, HCC PDOs exhibited an epithelial phenotype, including greater invadopodia formation and an irregular fibroblast‐like shape, which could be partially reversed by the addition of the SERPINE1 inhibitor PAI‐039 (10 μM) (Figure 4A). H.E. staining indicated that PDOs exhibited a pseudoglandular histological structure (Figure 4B). Immunofluorescence staining indicated three PDOs exhibited typical features of HCC, such as positive expression of AFP and barely EpCAM expression (Figure 4C–E). Meanwhile, sublethal heat treatment significantly stimulated SERPINE1 expression in all three PDOs, which could be attenuated by the addition of PAI‐039 (Figure 4C–F).

Figure 4SERPINE1 is upregulated in HCC organoid model under sublethal heat treatment. (A) The bright light microscope picture of three PDOs. (B) The H.E. staining of the three PDOs. (C–E) Immunofluorescence staining showed the level of AFP, EpCAM, and SERPINE1 in three PDOs. (F) The relative levels of SERPINE1 under the condition of 37, 46, and 46°C plus PAI‐039. “NS,” not significant,  ^∗^ *p*  < 0.05,  ^∗∗^ *p*  < 0.01,  ^∗∗∗^ *p*  < 0.001,  ^∗∗∗∗^ *p*  < 0.0001.

**Figure:**
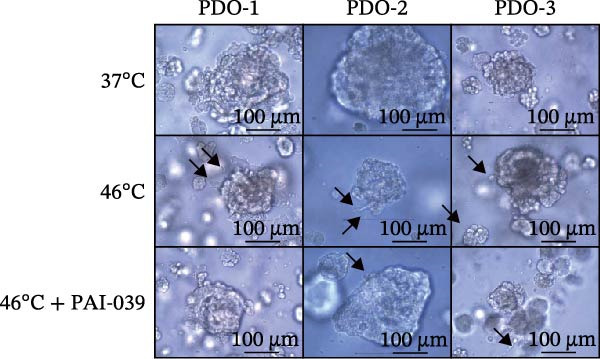
(A)

**Figure:**
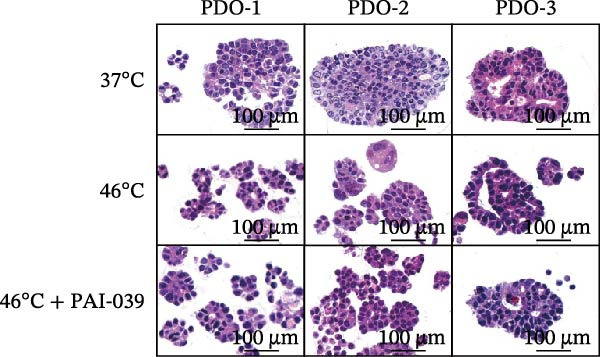
(B)

**Figure:**
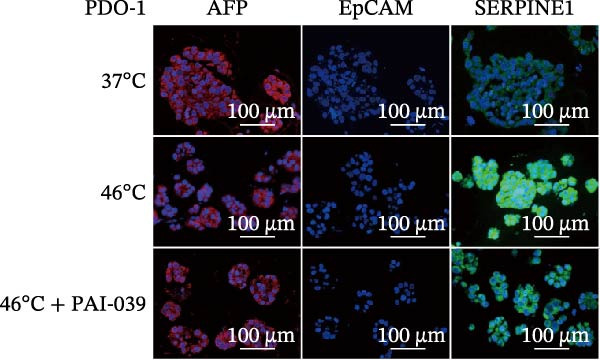
(C)

**Figure:**
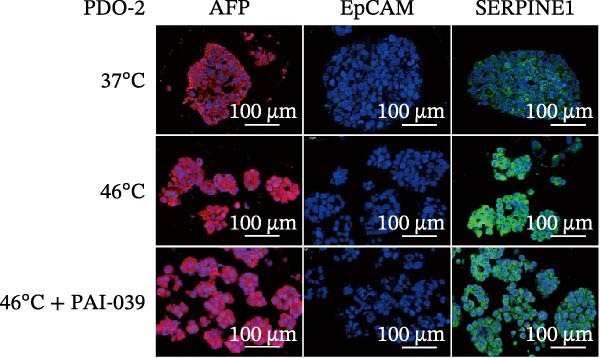
(D)

**Figure:**
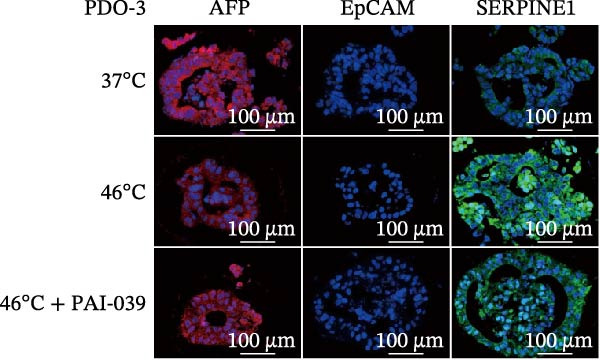
(E)

**Figure:**
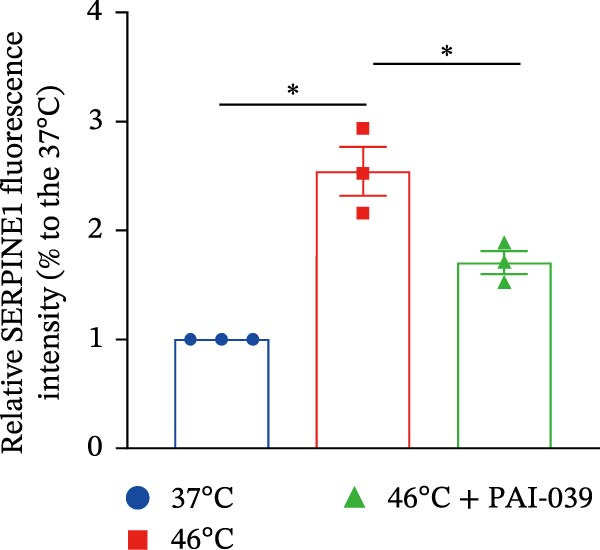
(F)

### 3.5. PAI‐039 Effectively Inhibits SERPINE1 Expression and Blocks Sublethal Heat Treatment‐induced Tumor Growth *In Vivo*

To test if inhibition of SERPINE1 with small molecule PAI‐039 can block sublethal heat treatment‐triggered tumor progression, we first established a sublethal‐heated HCCLM3 cell‐derived subcutaneous xenograft model. As Figure 5A shows, compared with the control group, the PAI‐039 treated group showed a significant decrease in tumor growth (Figure 5A,B). Additionally, analysis of CD31 immunostaining revealed a reduction in microvessel density within the PAI‐039 group, indicating an effective suppression of angiogenesis (Figure S1F,G). H.E. staining of the resected tumor tissues was also shown (Figure 5C). As expected, PAI‐039 treated group showed less expression of SERPINE1 than the control group detected by immunohistochemistry (Figure 5C). Notably, the expression of VEGFA was also decreased in the PAI‐039 treated group (Figure 5C), which aligns with the observed reduction in angiogenesis. Moreover, to parallel our findings with PAI‐039, subcutaneous tumors established from HCCLM3 cells with shSERPINE1 demonstrated a reduction in tumor progression that mirrored the effects observed with PAI‐039 treatment (Figure S1A–E), suggesting that knockdown of SERPINE1 phenocopies the results of pharmacological inhibition. On the other hand, we also observed that the model elucidated the regulatory mechanism of the METTL3/m6A/IGF2BP1/SERPINE1/VEGFA axis in angiogenesis in HCC (Figure 5D). These data collectively underscore the therapeutic potential of targeting SERPINE1, either by genetic silencing approaches such as shSERPINE1 or by pharmacological means with compounds like PAI‐039, in treating sublethal heat‐triggered HCC growth.

Figure 5PAI‐039 effectively inhibits SERPINE1 expression and blocks sublethal heat treatment‐induced tumor growth in vivo. (A) Ten female BALB/c nude mice received an injection of HCCLM3 with sublethal heat treatment. Five of them received saline and the other five received PAI‐039 every other day. Day 25 has reached is the end of the experiment; mice were sacrificed. (B) The tumor volume of the xenograft in both groups. (C) Representative H.E. staining, immunohistochemistry staining of SERPINE1 and immunohistochemistry staining of VEGFA in the control group and PAI‐039 group. (D) The working model of the METTL3/m6A/IGF2BP1/SERPINE1/VEGFA axis in sublethal heat treatment‐induced angiogenesis in HCC cells.

**Figure:**
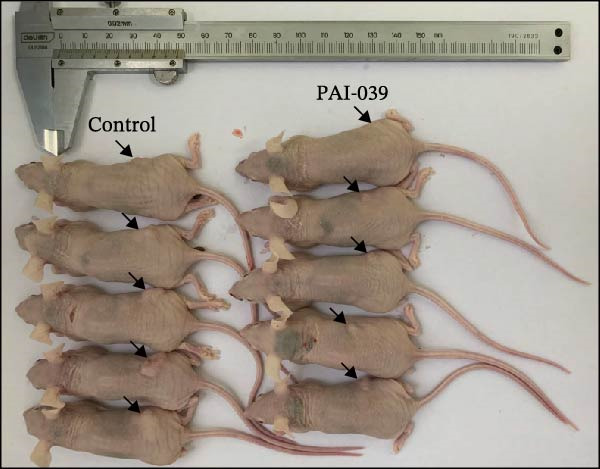
(A)

**Figure:**
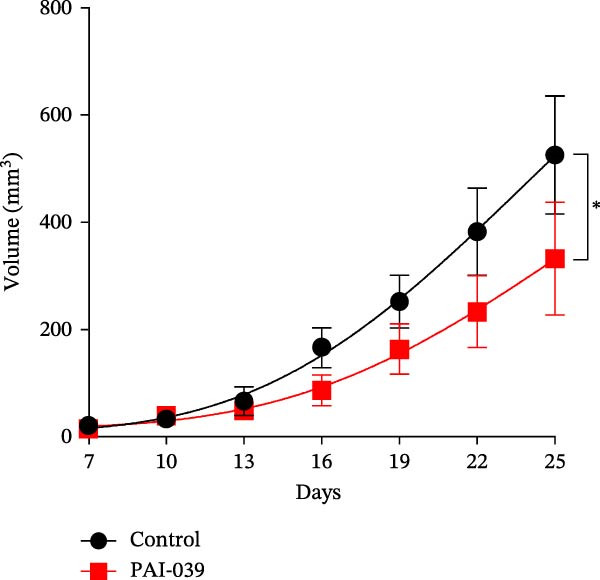
(B)

**Figure:**
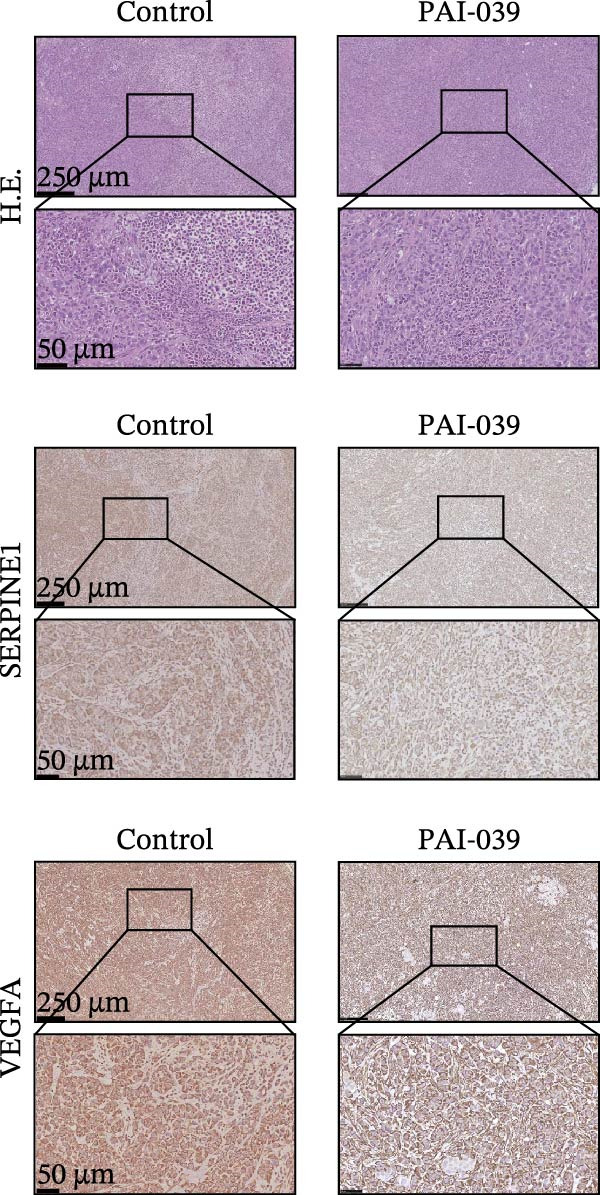
(C)

**Figure:**
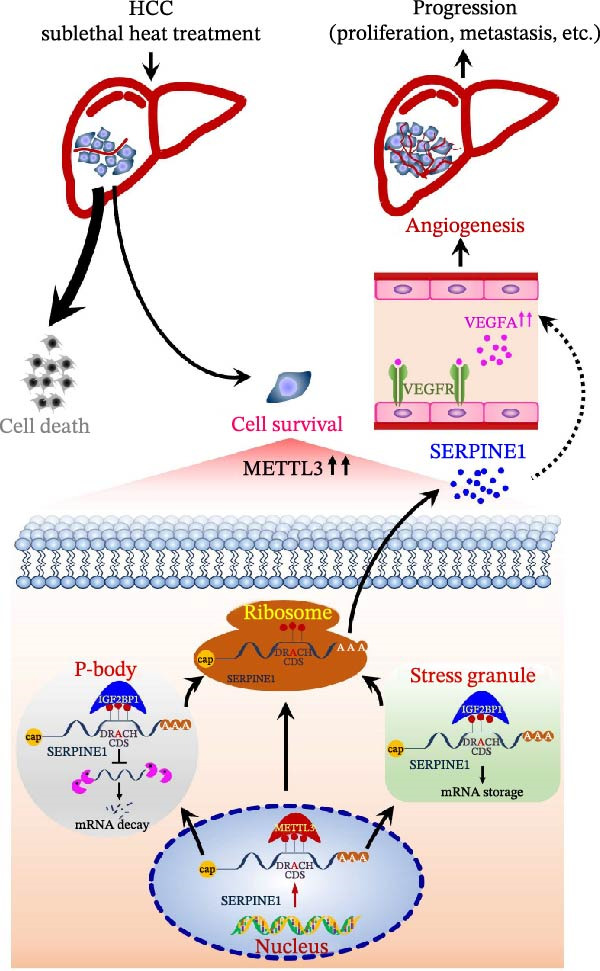
(D)

## 4. Discussion

Herein, we report that the SERPINE1/VEGFA axis contributes to sublethal heat ablation‐induced angiogenesis in HCC. We further demonstrate that heat stress activates a METTL3/IGF2BP1 signaling node, which positively regulates SERPINE1 mRNA stability in an m6A‐dependent manner, ultimately enhancing SERPINE1 protein expression and driving tumor progression (Figure 5D). To our knowledge, this is the first evidence linking m6A‐mediated epitranscriptomic regulation to angiogenesis in the context of thermal ablation.

Thermal ablation is a first‐line treatment for HCC [39], yet sublethal heating of residual tumors can paradoxically promote progression [40, 41]. Our study elucidates a novel mechanism underlying this adverse effect. While SERPINE1 and VEGFA are known proangiogenic factors [42, 43], their regulation by METTL3/m6A following thermal stress has not been previously described. The potent inhibition of heat‐induced angiogenesis and tumor growth by the SERPINE1 inhibitor PAI‐039, validated across cell lines, PDOs, and in vivo models, highlights the translational promise of this axis. Our work suggests that SERPINE1 inhibitors could serve as synergistic adjuvants to improve the efficacy of thermal ablation.

PDOs bridge the gap between traditional 2D cultures and in vivo models, offering greater clinical relevance [23–25]. Our use of HCC PDOs confirmed the heat‐induced upregulation of SERPINE1 and its inhibition by PAI‐039, strengthening the clinical applicability of our findings.

We acknowledge certain limitations in this study. While our functional data strongly support the proposed METTL3/m6A/IGF2BP1/SERPINE1/VEGFA axis, direct validation of m6A modification on SERPINE1 mRNA (e.g., via MeRIP‐qPCR) and physical interaction with IGF2BP1 (e.g., via RIP) would provide further mechanistic depth; these represent important directions for future research. Furthermore, while we selected SERPINE1 from a pool of angiogenesis‐related genes based on its dramatic upregulation and established biological role, the potential contributions of other heat‐responsive genes warrant independent investigation. Finally, although the cell lines used were from reputable sources and key findings were confirmed in PDOs, formal STR authentication was not performed during this study, a recognized best practice for future work.

In conclusion, our study reveals a novel METTL3/m6A/IGF2BP1/SERPINE1/VEGFA signaling axis that drives angiogenesis following sublethal thermal ablation in HCC. Targeting this axis, particularly with SERPINE1 inhibitors like PAI‐039, presents a promising strategy to suppress post‐ablation recurrence and improve patient outcomes.

## Funding

This work was supported by The fellowship of China National Postdoctoral Program for Innovative Talents (Grant BX20230083), the National Natural Science Foundation of China (Grant 82303564), the Youth Foundation of “Outstanding Resident Physician” Clinical Postdoctoral Program in Zhongshan Hospital (Grant 2024ZYYS‐031), and the Disciplinary Platform Construction and Development Fund of Zhongshan Hospital (Grant 2024XKPT22‐RC3).

## Ethics Statement

This study was approved by the Ethics Committee of Zhongshan Hospital, Fudan University.

## Conflicts of Interest

The authors declare no conflicts of interest.

## Supporting Information

Additional supporting information can be found online in the Supporting Information section.

## Supporting information


**Supporting Information** Figure S1. Knockdown of SERPINE1 blocks sublethal heat treatment‐induced tumor growth in vivo. (A) Five female BALB/c nude mice received an injection of negative control group of HCCLM3, and five female BALB/c nude mice received an injection of knockdown of SERPINE1 (shSERPINE1) of HCCLM3. Day 25 is the end of the experiment; mice were sacrificed. (B) The tumor volume of the xenograft in the control group and the shSERPINE1 group. (C) Representative H.E. staining in the control group and the shSERPINE1 group. (D) Representative immunohistochemistry staining of SERPINE1 in the control group and the shSERPINE1 group. (E) Representative immunohistochemistry staining of VEGFA in the control group and the shSERPINE1 group. (F) Representative immunohistochemistry staining of CD31 in the control group and the shSERPINE1 group. (G) Quantification of CD31 in the control group and the shSERPINE1 group. CD31 was clarified into the “Positive” group and the “Negative” group via Image J. “NS,” not significant,  ^∗^ *p* < 0.05,  ^∗∗^ *p* < 0.01,  ^∗∗∗^ *p* < 0.001, and  ^∗∗∗∗^ *p* < 0.0001.

## Data Availability

The data that support the findings of this study are available from the corresponding author upon reasonable request.
