# Supplementary material for: METTL3/m6A‐Dependent SERPINE1/VEGFA Axis Mediates Sublethal Heat‐Induced Angiogenesis in Hepatocellular Carcinoma
Source: Mediators Inflamm. 2026 Mar 24;2026:5133850. doi: 10.1155/mi/5133850 (PMC13140373; doi:10.1155/mi/5133850)
Supplement: Supplementary file 1 — Supporting Information Figure S1. Knockdown of SERPINE1 blocks sublethal heat treatment‐induced tumor growth in vivo. (A) Five female BALB/c nude mice received an injection of negative control group of HCCLM3, and five female BALB/c nude mice received an injection of knockdown of SERPINE1 (shSERPINE1) of HCCLM3. Day 25 is the end of the experiment; mice were sacrificed. (B) The tumor volume of the xenograft in the control group and the shSERPINE1 group. (C) Representative H.E. staining in the control group and the shSERPINE1 group. (D) Representative immunohistochemistry staining of SERPINE1 in the control group and the shSERPINE1 group. (E) Representative immunohistochemistry staining of VEGFA in the control group and the shSERPINE1 group. (F) Representative immunohistochemistry staining of CD31 in the control group and the shSERPINE1 group. (G) Quantification of CD31 in the control group and the shSERPINE1 group. CD31 was clarified into the “Positive” group and the “Negative” group via Image J. “NS,” not significant, ∗ p < 0.05, ∗∗ p < 0.01, ∗∗∗ p < 0.001, and ∗∗∗∗ p < 0.0001. [file MI-2026-5133850-s001.pdf]

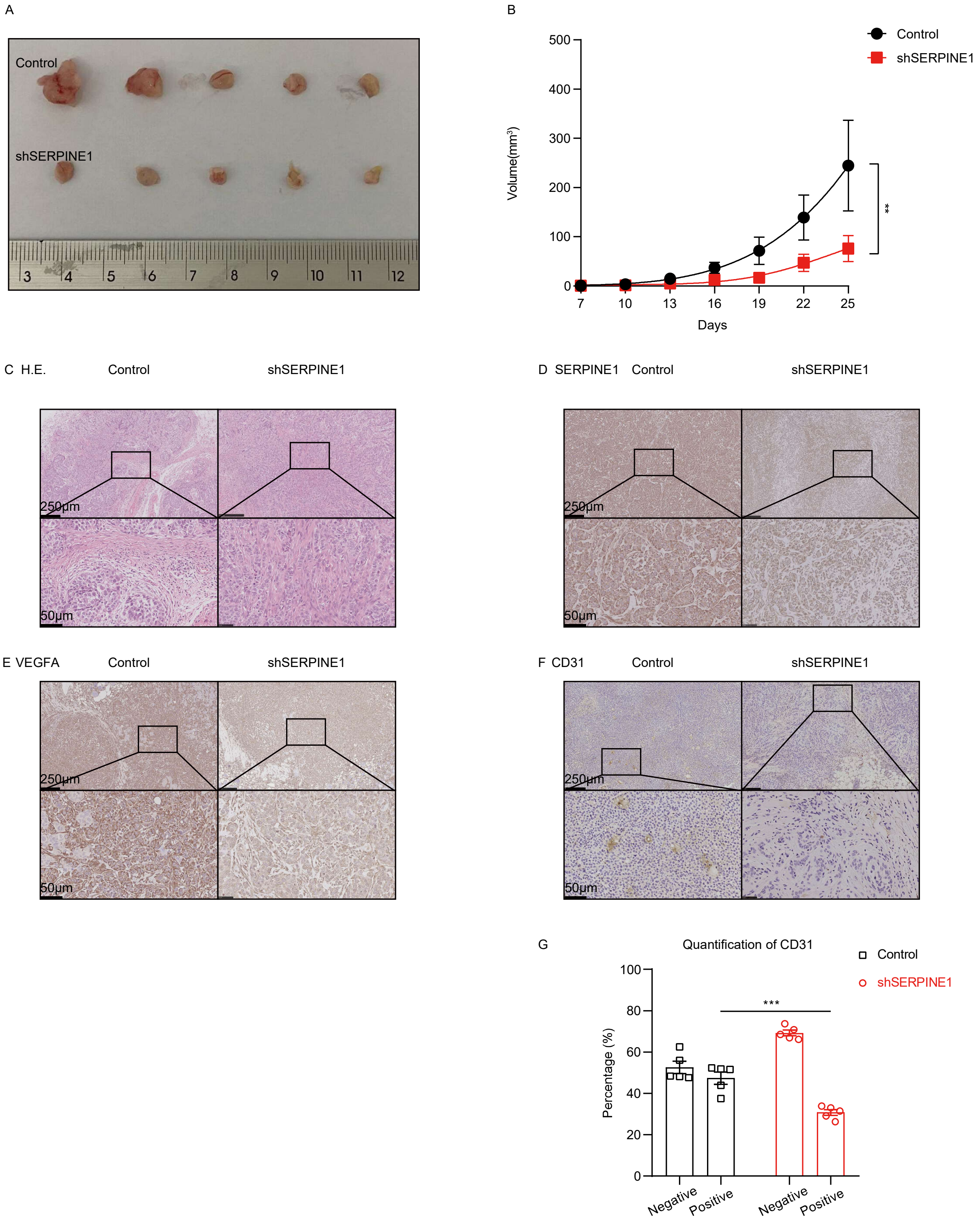
A
Control
shSERPINE1
3 4 5 6 7 8 9 10 11 12
B
Control
shSERPINE1
Volume(mm³)
500
400
300
200
100
0
7 10 13 16 19 22 25
Days
**
C H.E.
Control
shSERPINE1
250μm
50μm
D SERPINE1
Control
shSERPINE1
250μm
50μm
E VEGFA
Control
shSERPINE1
250μm
50μm
F CD31
Control
shSERPINE1
250μm
50μm
G
Quantification of CD31
Control
shSERPINE1
Percentage (%)
100
80
60
40
20
0
***
Negative
Positive
Negative
Positive
